# First‐line immune‐based combination therapies for advanced non‐small cell lung cancer: A Bayesian network meta‐analysis

**DOI:** 10.1002/cam4.4405

**Published:** 2021-11-07

**Authors:** Ziyang Mao, Panpan Jiang, Yajuan Zhang, Yanlin Li, Xiaohui Jia, Qinyang Wang, Min Jiao, Lili Jiang, Yuan Shen, Hui Guo

**Affiliations:** ^1^ Department of Medical Oncology The First Affiliated Hospital of Xi’an Jiaotong University Xi’an Shaanxi P.R. China; ^2^ Department of Epidemiology and Biostatistics School of Public Health Xi'an Jiaotong University Health Science Center Xi’an Shaanxi P.R. China; ^3^ Key Laboratory of Environment and Genes Related to Diseases Xi'an Jiaotong University Ministry of Education of China Xi'an Shaanxi P.R. China; ^4^ Centre for Translational Medicine The First Affiliated Hospital of Xi'an Jiaotong University Xi'an Shaanxi P.R. China

**Keywords:** efficacy, immune‐based combination therapies, network meta‐analysis, non‐small cell lung cancer, safety

## Abstract

**Background:**

Immune‐based combination therapies have revolutionized the first‐line treatment for advanced non‐small cell lung cancer (NSCLC). However, for the efficacy and safety, the best treatment option is still uncertain.

**Methods:**

We conducted a Bayesian network meta‐analysis of randomized controlled trials (RCTs) to evaluate first‐line immune‐based combination therapies for advanced NSCLC.

**Results:**

Fourteen trials involving 8467 patients were included. For the programmed cell death‐ligand 1 (PD‐L1) expression non‐selective patients, there were no significant differences among all the treatment modes for overall survival (OS), but the ranking profiles indicated that Immunotherapy + Immunotherapy + Chemotherapy (IO + IO + Chemo) was most likely to be the best mode (probability = 68%). Immunotherapy + Immunotherapy + Anti‐angiogenic therapy + Chemotherapy (IO + Anti‐angio + Chemo) was significantly better than most other treatment modes for progression‐free survival (PFS) with better objective response rate (ORR) and more obvious grade ≥3 treatment‐related adverse events (TRAEs). In PD‐L1‐high cohort, IO + Anti‐angio + Chemo seemed to be the best mode for OS, PFS, and ORR according to the ranking profiles. In PD‐L1‐intermediate and PD‐L1‐negative cohort, IO + IO + Chemo was inclined to be ranked first for prolonging OS (probability = 78%; 37%) and IO + Anti‐angio + Chemo was most likely to provide best PFS (probability = 96%; 100%).

**Conclusion:**

IO + IO + Chemo has great potential to improve the OS regardless of histology type, especially in PD‐L1‐intermediate and PD‐L1‐negative cohort. IO + Anti‐angio + Chemo shows great superiority in improving the short‐term survival accompanied by increasing grade ≥3 TRAEs.

## Graphical abstract

IO + IO + Chemo has great potential to be recommended to improve OS regardless of the histology type especially in PD‐L1‐intermediate and PD‐L1‐negative cohort. IO + Anti‐angio + Chemo shows great superiority in improving the short‐term survival accompanied by relatively increasing TRAEs. Our findings are of great significance for the first‐line treatment of advanced NSCLC and provide reference for clinicians and patients to achieve precise therapy.

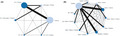

## 1 INTRODUCTION

Lung cancer is the major cause of cancer‐related mortality all over the world in the 21st century.^1^ Non‐small cell lung cancer (NSCLC) is the most common type, which is generally diagnosed at an advanced stage.^2^ As for patients without sensitive gene mutations, they are unable to benefit from targeted therapy, making the choices of treatment full of passivity for them. Recently, immunotherapy, represented by immune checkpoint inhibitors (ICIs), has become a burgeoning treatment option for these patients and revolutionized the treatment of advanced NSCLC.^3^ However, the proportion of patients with primary response to immunotherapy is low. The overall response rate is 10%–20% when PD‐L1 level is not considered.^4^ Combination therapy can generate synergistic effects, which will produce antitumor effects in more patients with ineffective monotherapy.^5^ Therefore, numerous treatment modes of immune‐based combination therapies have been emerging. At present, in the field of immune‐based combination therapies for advanced NSCLC patients without sensitive gene mutations, the main modes include: Immunotherapy + Chemotherapy (IO + Chemo), Immunotherapy + Immunotherapy (IO + IO), Immunotherapy + Immunotherapy + Chemotherapy (IO + IO + Chemo), and Immunotherapy + Anti‐angiogenic therapy + Chemotherapy (IO + Anti‐angio + Chemo). How to choose these modes wisely is a clinical puzzle.

Among IO + Chemo mode, Pembrolizumab + Chemotherapy (Pembro + Chemo) and Atezolizumab + Chemotherapy (Atezo + Chemo) have been approved in the first‐line treatment for advanced NSCLC patients without sensitive gene mutations according to promising results of KEYNOTE 407,^6^ KEYNOTE 189,^7^ and IMpower 132.^8^ IO + IO mode is initiated by Checkmate 227,^9^ providing a new choice of “non‐chemotherapy” for the first‐line treatment for advanced NSCLC. No matter what the expression level of PD‐L1 is, IO + IO mode, represented by Nivolumab + Ipilimumab (Nivo + Ipi), can achieve significant overall survival (OS) improvement compared with chemotherapy. On the basis of IO + IO mode, Checkmate 9LA^10^ creatively added two cycles of concurrent chemotherapy, forming the IO + IO + Chemo mode. The results also confirm that this mode can bring greater survival benefit to patients compared with chemotherapy (median OS: 15.6 months vs. 10.9 months, HR = 0.66, *p* = 0.02). IMpower150^11^ is the first phase III study to confirm that metastatic non‐squamous NSCLC can significantly benefit more from IO + Anti‐angio + Chemo mode, represented by Atezolizumab + Bevacizumab + Chemotherapy (Atezo + Beva + Chemo), as the first‐line treatment compared with standard Anti‐angio + Chemo, namely Bevacizumab + Chemotherapy (Beva + Chemo) (median OS: 19.5 months vs. 14.7 months, HR = 0.80, *p* = 0.01). With the emergence of numerous immune‐based combination therapies better than chemotherapy‐based treatments emerging, how to choose the best treatment option has been attracted more and more attentions. Unfortunately, there is no study on the direct comparisons of the above specific combination treatment modes. Therefore, it is still a puzzle for clinicians to choose the treatment mode wisely in order to bring huge therapeutic effects and controllable treatment‐related adverse events (TRAEs).

There are several existing meta‐analysis indirectly comparing the efficacy and safety of a variety of specific treatment regimens.^12^, ^13^, ^14^ However, they failed to comprehensively compare the differences among the above four treatment modes based on immunotherapy within a large framework. Based on the above context, we designed and completed this network meta‐analysis to compare the efficacy and safety of existing first‐line immune‐based combination therapies for advanced NSCLC.

## 2 MATERIALS AND METHODS

We conducted this network meta‐analysis in accordance with the preferred reporting items for systematic reviews and meta‐analyses (PRISMA) guidelines.^15^ A protocol was designed for this network meta‐analysis and registered in the Prospective Register of Systematic Reviews (PROSPERO CRD42021224341).

### 2.1 Search strategy

PubMed, Embase, and the Cochrane Central Register of Controlled Trials were the sources of eligible randomized controlled trials (RCTs). We searched for studies published in English before 1 October 2020, using the keywords including pembrolizumab, atezolizumab, PD‐1, PD‐L1, NSCLC, RCTs, etc. International conference, such as American Society of Clinical Oncology (ASCO), European Society of Medical Oncology (ESMO), American Association for Cancer Research (AACR), and World Conference on Lung Cancer (WCLC) were also taken into account to avoid the loss of information. The latest study with updated data was included when duplicate studies existed. Table S1 presents the detailed search strategy. Two reviewers (Z.M. and P.J.) set eligibility criteria and checked the studies independently.

### 2.2 Inclusion and exclusion criteria

A study was included when met all the following criteria: (a) histologically confirmed previously untreated NSCLC without sensitive gene mutations; (b) phase II/III RCTs with primary endpoints, such as OS, progression‐free survival (PFS), or objective response rate (ORR); and (c) the intervention group was treated with any immune‐based combination therapy, whereas the control group was treated with non‐immunotherapy, such as chemotherapy or chemotherapy plus anti‐angiogenic therapy.

The corresponding exclusion criteria were as follows: (a) trials involving pretreated patients; (b) designed as observational studies; (c) lack of related data; (d) published as meta‐analysis, editorials, reviews, and case reports; and (e) single‐arm or dosage‐finding studies.

### 2.3 Data extraction and risk of bias assessment

Two authors (Z.M. and P.J.) reviewed the retrieved studies in detail and extracted data independently. The following items for each included trial were extracted: trial name, publication year, phase of trials, number and characteristics of patients, treatments, and survival data (OS, PFS, ORR, and grade ≥3 TRAEs). Discrepancies were adjudicated by a superior investigator (H.G.).

The Cochrane Risk of Bias Tool^16^ was adopted by two independent authors (Y.Z. and Y.L.) to assess the quality of included studies. Following items were assessed: random sequence generation, allocation concealment, blinding of participants and personnel, blinding of outcome assessment, incomplete outcome data, selective outcome reporting, and other sources of bias. Discrepancies were resolved via discussion among all researchers.

### 2.4 Statistical analysis

In this study, we combined all the direct and indirect evidence to compare the efficacy and safety of different treatment modes and regimens. Hazard ratios (HRs) were reported for OS and PFS and odds ratios (ORs) were reported for ORR and grade ≥3 TRAEs with the corresponding 95% confidence interval (CI). OS and PFS were primary outcomes. Secondary outcomes were ORR and grade ≥3 TRAEs.

In STATA (version 14), we conducted network plots of both treatment modes and treatment regimens to clarify relationship of direct comparisons and indirect comparisons among these treatment options in the included studies.

For Bayesian network‐meta analysis (NMA), Win BUGS (version 14) and gemtc (version 0.14.3) were applied to pool indirect evidence of OS, PFS, ORR, and grade ≥3 TRAEs in fixed‐effect model. We used non‐informative uniform and normal prior distributions to fit the model. For OS and PFS effects, three chains and 150,000 sample iterations were generated with 100,000 burn‐ins and a thinning interval of 10. For ORR and grade ≥3 TRAEs, 50,000 sample iterations were generated with 20,000 burn‐ins and a thinning interval of 10. Moreover, we identified the probability of each treatment options to be ranked the first. The ranking profile was used to provide a simple treatment rankings. For each outcome, the probability equaled 1 if the treatment was certain to be ranked the first and 0 if it was certain to be to be ranked the last.

For traditional meta‐analysis, direct evidence was pooled in pair‐wise meta‐analysis (PWMA) using RevMan (version 5.4). The *χ* ^2^ test and *I* ^2^ statistic were applied to estimate heterogeneity. If *p* < 0.10 for the *χ* ^2^ test or *I* ^2^ > 50%, we recognized heterogeneity was great. The random effects model was adopted for potential heterogeneity in these studies.

## 3 RESULTS

### 3.1 Eligible studies and characteristics

**FIGURE 1:**
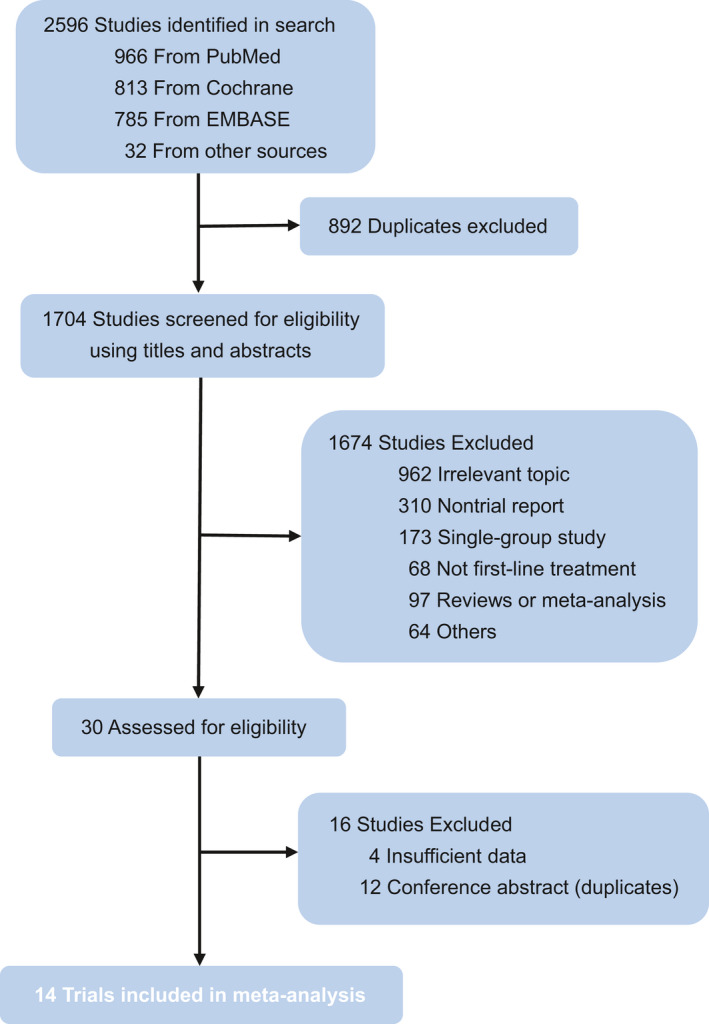
Flow chart of study selection. Based on the inclusion and exclusion criteria, 14 trials were included in this study

**FIGURE 2:**
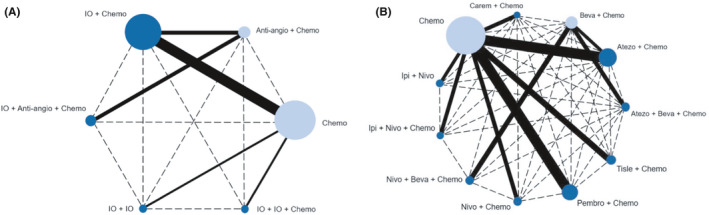
Network structure for all the included trials. Network structure according to (A) treatment modes and (B) treatment regimens. Each circular node represents a treatment type (dark blue represents the main object for analysis of study and light blue represents the object that is only used for transmission and not for main analysis). The circle size is proportional to the total number of patients. The width of solid lines is proportional to the number of studies performing head‐to‐head comparisons in the same study. The dotted lines represent indirect comparisons. Anti‐angio + Chemo, Anti‐angiogenic therapy + Chemotherapy; Chemo, Chemotherapy; IO + Anti‐angio + Chemo, Immunotherapy + Anti‐angiogenic therapy + Chemotherapy; IO + Chemo, Immunotherapy + Chemotherapy; IO + IO, Immunotherapy + Immunotherapy; IO + IO + Chemo, Immunotherapy + Immunotherapy + Chemotherapy

We identified 2596 records through the initial search strategy. The detail of the search criteria is shown in Table S1. Eventually, a total of 14 trials^6^, ^8^, ^9^, ^10^, ^11^, ^17^, ^18^, ^19^, ^20^, ^21^, ^22^, ^23^, ^24^, ^25^ were included, with 9454 participants enrolled. The detail of the selection process is shown in Figure 1. These original studies were published in well‐known journals or international conferences. Among these trials, only Checkmate 227 included part 1 and part 2. The networks are presented in Figure 2. The characteristics of all included studies are listed in Table 1, and some additional information is presented in Table 2. The Cochrane Risk of Bias Tool for bias assessment of included studies is shown in Figure S1.

**TABLE 1:** Baseline characteristics of RCTs included in the network meta‐analysis of advanced non‐small cell lung cancer patients treated with immune‐based combination therapies

Trial	Publication	Trial phase	Stage	Histology	Regimen(s)	Maintenance therapy	Patients (*n*)
IMpower 130	Lan Onco 2019	III	IV	Non‐squamous	Atezolizumab 1200 mg + carboplatin AUC 6 + nab‐paclitaxel 100 mg/m^2^ q3w for four or six cycles	Atezolizumab 1200 mg q3w until PD	451
					Carboplatin AUC 6 + nab‐paclitaxel 100 mg/m^2^ q3w for four or six cycles	BSC or pemetrexed 500 mg/m^2^ q3w	228
IMpower 131	J Thorac Onco 2020	III	IV	Squamous	Atezolizumab 1200 mg + carboplatin AUC 6 q3w + nab‐paclitaxel 100 mg/m^2^ q1w for four or six cycles	Atezolizumab 1200 mg q3w until PD	343
					Carboplatin AUC 6 q3w + nab‐paclitaxel 100 mg/m^2^ q1w for four or six cycles	Best Supportive Care until PD	340
IMpower 132	ESMO 2020	III	IV	Non‐squamous	Atezolizumab 1200 mg + pemetrexed 500 mg/m^2^ + (cisplatin 75 mg/m^2^ or carboplatin AUC 6) q3w × 4 or 6	Atezolizumab 1200 mg + pemetrexed 500 mg/ m^2^ q3w	292
					Pemetrexed 500 mg/m^2^ + (cisplatin 75 mg/m^2^ orcarboplatin AUC 5) q3w × 4 or 6	Pemetrexed 500 mg/m^2^ q3w	286
KEYNOTE 189	J Clin Oncol 2020	III	IV	Non‐squamous	Pembrolizumab 200 mg + pemetrexed 500 mg/m^2^ + (cisplatin 75 mg/m^2^ orcarboplatin AUC 6) q3w × 4	Pembrolizumab 200 mg q3w ≤x31 + pemetrexed 500 mg/m^2^ q3w	410
					Placebo + pemetrexed 500mg/m^2^ + (cisplatin 75 mg/m^2^ or carboplatin AUC 5) q3w × 4	Placebo + pemetrexed 500 mg/m^2^ q3w	206
KEYNOTE 407	J Thorac Oncol 2020	III	IV	Squamous	Pembrolizumab 200 mg + carboplatin AUC 6 q3w + paclitaxel 200 mg/m^2^ q3w or nab‐paclitaxel 100 mg/m^2^ q1w for four cycles	Pembrolizumab 200 mg q3w ≤x31	278
					Placebo + carboplatin AUC 6 q3w + paclitaxel 200 mg/m^2^ q3w or nab‐paclitaxel 100 mg/m^2^ q1w for four cycles	Placebo q3w ≤x31	281
KEYNOTE 021G	AACR 2019	II	III, IV	Non‐squamous	Pembrolizumab 200 mg + pemetrexed 500 mg/m^2^ + AUC 6 q3w for four cycles	Pembrolizumab 200 mg q3w ≤ x32	60
					Pemetrexed 500 mg/m^2^ + AUC 6 q3w for four cycles	Pemetrexed 500 mg/m^2^	62
CAMEL	WCLC 2019	III	IV	Non‐squamous	Camrelizumab 200 mg + carboplatin AUC 5 + pemetrexed 500 mg/m^2^ q3w for 4–6 cycles	Camrelizumab 200 mg + pemetrexed 500 mg/m^2^ q3w until PD	205
					Carboplatin AUC 5 + pemetrexed 500 mg/m^2^ q3w for 4–6 cycles	Pemetrexed 500 mg/m^2^ q3w until PD	207
RATIONALE 304	ESMO 2020	III	III, IV	Non‐squamous	Tislelizumab 200 mg + (cisplatin 75 mg/m^2^ orcarboplatin AUC 6) + pemetrexed 500 mg/m^2^ q3w for 4–6 cycles	Tislelizumab 200 mg + pemetrexed 500 mg/m^2^ q3w until PD	223
					(cisplatin 75 mg/m^2^ orcarboplatin AUC 6) + pemetrexed 500 mg/m^2^ q3w for 4–6 cycles	Pemetrexed 500 mg/m^2^ q3w until PD	111
RATIONALE 307	ESMO 2020	III	III, IV	Squamous	Tislelizumab 200 mg + paclitaxel 175 mg/m^2^ + carboplatin AUC5 q3w for 4–6 cycles	Tislelizumab 200 mg q3w until PD	120
					Paclitaxel 175 mg/m^2^ + carboplatin AUC5 q3w for 4–6 cycles	None	121
NCT01285609	J Clin Oncol 2017	III	IV,recurrent	Squamous	Ipilimumab 10 mg/kg + paclitaxel 175 mg/m^2^ for four cycles + carboplatin AUC5 q3w for six cycles	Ipilimumab 10 mg/kg q12w until PD or unacceptable toxicity	479
					Paclitaxel 175 mg/m^2^ + carboplatin AUC5 q3w for six cycles	Placebo q12w until PDor unacceptable toxicity	361
CheckMate 227 Part 2	ESMO‐IO 2019	III	IV,recurrent	Squamous and Non‐squamous	Nivolumab 360 mg + histology‐based chemo q3w (chemo≤4 cycles)	None	377
					Histology‐based chemo q3w (chemo≤4 cycles)	Pemetrexed 500 mg/m^2^q3w (non‐squamousonly)	378
CheckMate 227 Part 1	ASCO 2020	III	IV,recurrent	Squamous and Non‐squamous	Nivolumab 3 mg/kg q2w + ipilimumab 1 mg/kg q6w	None	583
					Histology‐based chemo	Pemetrexed 500 mg/m^2^q3w (non‐squamous only)	583
CheckMate 9LA	ASCO 2020	III	IV	Squamous and Non‐squamous	Nivolumab 360 mg q3w + ipilimumab 1 mg/kgq6w + chemotherapy for two cycles	None	361
					Chemotherapy for four cycles	Pemetrexed (non‐squamousonly)	358
IMpower150	AACR 2020	III	IV	Non‐squamous	Atezolizumab 1200 mg + carboplatin AUC 6 + paclitaxel 200 mg/m^2^ q3w for four or six cycles	Atezolizumab 1200 mg q3w until PD	402
					Atezolizumab 1200 mg + carboplatin AUC 6 + paclitaxel 200 mg/m^2^ q3w for four or six cycles + bevacizumab 15 mg/kg q3w	Atezolizumab 1200 mg + bevacizumab 15 mg/kg IV q3w until PD	400
					Carboplatin AUC 6 + paclitaxel 200 mg/m^2^ q3w for four or six cycles + bevacizumab 15 mg/kg q3w	Bevacizumab 15 mg/kg q3w until PD	400
TASUKI‐52	ESMO 2020	III	III, Ⅳ	Non‐squamous	Nivolumab 360 mg + Carboplatin AUC 6 + paclitaxel 200 mg/m^2^ q3w for four or six cycles + bevacizumab 15 mg/kg q3w	Nivolumab 360 mg + bevacizumab 15 mg/kg q3w until PD	273
					Placebo + carboplatin AUC 6 + paclitaxel 200 mg/m^2^ q3w for four or six cycles + bevacizumab 15 mg/kg q3w	Placebo + bevacizumab 15 mg/kg q3w until PD	275

Abbreviations: AACR, American Association for Cancer Research; ASCO, American Society of Clinical Oncology congress; ESMO, conference of European Society for Medical Oncology congress; ESMO‐IO, conference of European Society for Medical Oncology congress, Immuno‐Oncology Congress; J Clin Oncol, Journal of Clinical Oncology; J Thorac Oncol, Journal of Thoracic Oncology; Lan Onco, Lancet Oncology; WCLC, World conference on lung cancer.

**TABLE 2:** Study and demographical characteristics of included RCTs included in the network meta‐analysis of advanced non‐small cell lung cancer patients treated with immune‐based combination therapies

Trial	Male (%)		Age (median)		Non‐squamous cell carcinoma (%)		Smoker (%)		ECOG 0 (%)		Asia (%)		Liver metastases (%)	
	Experiment	Control	Experiment	Control	Experiment	Control	Experiment	Control	Experiment	Control	Experiment	Control	Experiment	Control
IMpower 130	266 (59.0)	134 (58.8)	64	65	451 (100)	228 (100)	403 (89.4)	211 (92.5)	189 (42.0)	91 (39.9)	12 (2.7)	3 (1.3)	69 (15)	31 (14)
IMpower 131	279(81.0)	278 (82.0)	65	65	0	0	311 (91.0)	316 (93.0)	115 (34.0)	110 (32.0)	41 (12.0)	37 (11.0)	70 (20.4)	69 (20.3)
IMpower 132	192 (65.8)	192 (67.1)	64	63	292 (100)	286(100)	255 (87.3)	256 (89.5)	126 (43.2)	114 (40.1)	71 (24.3)	65 (22.7)	0	0
KEYNOTE 189	254 (62.0)	109 (52.9)	65	63.5	410 (100)	206 (100)	362 (88.3)	181 (87.9)	186 (45.4)	80 (38.8)	4 (1.0)	6 (2.9)	66 (16.1)	49 (23.8)
KEYNOTE 407	220 (79.1)	235 (83.6)	65	65	0	0	256 (92.1)	262 (93.2)	73 (26.3)	90 (32.0)	54 (19.4)	52 (18.5)	0	0
KEYNOTE 021G	22 (37.0)	26 (41.0)	62.5	63.2	60 (100)	63 (100)	45 (75.0)	54 (86.0)	24 (40.0)	29 (46.0)	5 (8.0)	5 (8.0)	0	0
CAMEL	146 (71.2)	149 (72.0)	59	61	205 (100)	207 (100)	NA	NA	48 (23.4)	36 (17.5)	NA	NA	0	0
RATIONALE 304	168 (75.3)	79 (71.2)	60	61	223 (100)	111 (100)	147 (65.9)	66 (59.4)	54 (24.2)	24 (21.6)	NA	NA	20 (9.0)	17 (15.3)
RATIONALE 307	112 (94.1)	111 (91.7)	63	62	0	0	107 (89.9)	98 (81.0)	22 (18.5)	32 (26.4)	NA	NA	15 (12.6)	14 (11.6)
NCT01285609	326 (84.0)	309(85.6)	64	64	0	0	383(98.7)	317(87.8)	135(34.8)	124(34.3)	NA	NA	NA	NA
CheckMate 227 Part 1	393 (67.4)	385 (66.0)	64	64	419 (71.9)	421 (72.2)	497 (85.2)	499 (85.6)	204 (35.0)	191 (32.8)	NA	NA	NA	NA
CheckMate 227 Part 2	393 (67.4)	385 (66.0)	64	64	419 (71.9)	421 (72.2)	497 (85.2)	499 (85.6)	204 (35.0)	191 (32.8)	NA	NA	NA	NA
CheckMate 9LA	252 (70.0)	252 (70.0)	65	65	249 (69.0)	247 (69.0)	314 (87.0)	308 (86.0)	112 (31.0)	111 (31.0)	NA	NA	69 (19)	86 (24)
IMpower150	240 (60.0)	239 (59.8)	63	63	400 (100)	400 (100)	318 (79.5)	323 (80.8)	159 (40.1)	179 (45.1)	56 (14.0)	46 (11.5)	53 (13.2)	57 (14.2)
TASUKI−52	163 (91.1)	164 (92.1)	64	62	179 (100)	178 (100)	155 (86.6)	147 (82.6)	30 (16.8)	22 (12.4)	275 (100)	275 (100)	NA	NA

Abbreviation: NA, not available.

### 3.2 Primary outcomes

#### 3.2.1 Efficacy and safety of treatment modes in the whole population

**FIGURE 3:**
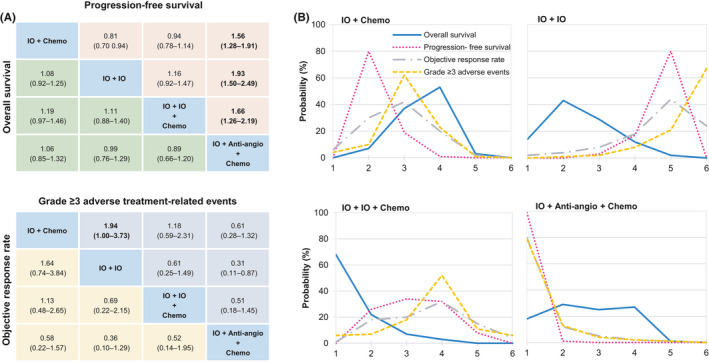
Efficacy and safety for treatment modes for the whole population. (A) Pooled estimates of the network meta‐analysis. Hazard ratios less than 1 and odds ratios more than 1 favor the former treatment. Significant results are in bold. (B) Bayesian ranking profiles of comparable treatment modes on efficacy and safety. Profiles indicate the probability of each comparable treatment being ranked from first to last on overall survival, progression‐free survival, objective response rate, and Grade ≥3 adverse events. We did not show the analysis for Anti‐angio + Chemo and Chemo. Anti‐angio + Chemo, Anti‐angiogenic therapy + Chemotherapy; Chemo, Chemotherapy; IO + Anti‐angio + Chemo, Immunotherapy + Anti‐angiogenic therapy + Chemotherapy; IO + Chemo, Immunotherapy + Chemotherapy; IO + IO, Immunotherapy + Immunotherapy; IO + IO + Chemo, Immunotherapy + Immunotherapy + Chemotherapy

The results of indirect comparisons of treatment modes are presented in Figure 3A. For PFS, the other three treatment modes were inferior to IO + Anti‐angio + Chemo (HR range: 1.56–1.93), which were in accordance with the results of Bayesian ranking profiles (Figure 3B) that IO + Anti‐angio + Chemo was most likely to the best treatment mode for PFS (probability = 99%). From the perspective of OS, there seemed to be no significant differences among the treatment modes, but the ranking profiles (Figure 3B) indicated that IO + IO + Chemo was most likely to be the best mode for increasing OS (probability = 68%). For ORR and grade ≥3 TRAEs, no significant differences were detected in these modes. The ranking profiles (Figure 3B) indicated that IO + Anti‐angio + Chemo had the highest opportunity to benefit ORR and confront grade ≥3 TRAEs among all treatment modes (probability = 58%; 77%). The detailed probability distribution of ranking is shown in Table S2.

#### 3.2.2 Efficacy and safety of treatment regimens in the whole population

**FIGURE 4:**
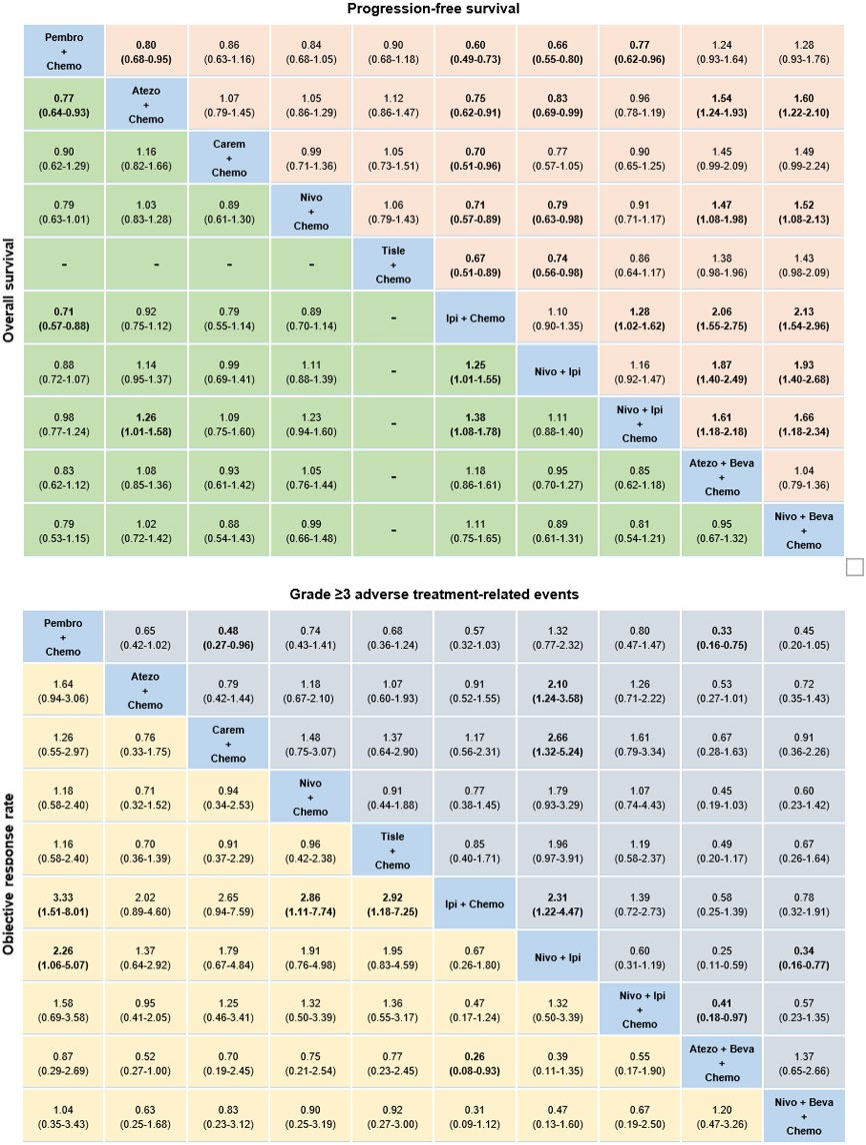
Efficacy and safety for treatment regimens for the whole population. Pooled estimates of the network meta‐analysis. Hazard ratios less than 1 and odds ratios more than 1 favor the former treatment. Significant results are in bold. We did not show the analysis results for Beva + Chemo and Chemo. Atezo + Beva + Chemo, Atezolizumab + Bevacizumab + Chemotherapy; Carem + Chemo, Camrelizumab + Chemotherapy; Ipi + Chemo, Ipilimumab + Chemotherapy; Nivo + Beva + Chemo, Nivolumab + Bevacizumab + Chemotherapy; Nivo + Ipi, Nivolumab + Ipilimumab; Nivo + Ipi + Chemo, Nivolumab + Ipilimumab + Chemotherapy; Pembro + Chemo, Pembrolizumab + Chemotherapy; Tisle + Chemo, Tislelizumab + Chemotherapy

We also made an analysis of the treatment regimens for the whole population. For OS (Figure 4), Pembro + Chemo, Nivo + Ipi, and Nivolumab + Ipilimumab + Chemotherapy (Nivo + Ipi + Chemo) performed significantly better OS than Ipilimumab + Chemotherapy (Ipi + Chemo). Moreover, Pembro + Chemo and Nivo + Ipi + Chemo were both superior to Nivo + Ipi significantly and no significant difference was found between Pembro + Chemo and Nivo + Ipi + Chemo. But from the ranking profiles (Figure S2), we found that Pembro + Chemo was most likely to be the best regimen (probability = 39%).

For PFS (Figure 4), no significant difference was found between Atezo + Beva + Chemo and Nivo + Beva + Chemo in IO + Anti‐angio + Chemo mode and both of them showed significantly greater survival improvement compared with most other regimens except Pembro + Chemo, Camrelizumab + Chemotherapy (Carem + Chemo), and Tislelizumab + Chemotherapy (Tisle + Chemo). The ranking profiles (Figure S2) indicated Nivo + Beva + Chemo had the highest chance to be ranked first in improving PFS (probability = 58%).

In the matter of ORR (Figure 4), Pembro + Chemo, Nivo + Chemo, Tisle + Chemo, and Atezo + Beva + Chemo were significantly superior to Ipi + Chemo in different degrees. Besides, Pembro + Chemo performed significantly better than Nivo + Ipi (OR = 2.26, 95% CI: 1.06–5.07) exclusively. As shown in the ranking profiles (Figure S2), Atezo + Beva + Chemo was most likely to be ranked first to offer best ORR (probability = 43%).

For grade ≥3 TRAEs (Figure 4), Atezo + Chemo, Carem + Chemo, Ipi + Chemo, Nivo + Beva + Chemo, and Atezo + Beva + Chemo tended to increase tolerability compared to Nivo + Ipi significantly. The ranking profiles (Figure S2) indicated that Nivo + Ipi was probably the least toxic regimen (probability = 45%) and Atezo + Beva + Chemo had a potential to cause more toxicity than other regimens (probability = 65%). Besides, Pembro + Chemo was significantly safer than Carem + Chemo (OR = 0.48, 95% CI: 0.27–0.96) and Atezo + Beva + Chemo (OR = 0.33, 95% CI: 0.16–0.75).

The detailed probability distribution of ranking about efficacy and safety of treatment regimens in the whole population is shown in Table S3.

### 3.3 Subgroup analysis

#### 3.3.1 Efficacy analysis of treatment modes according to PD‐L1 expression

##### PD‐L1‐high cohort

**FIGURE 5:**
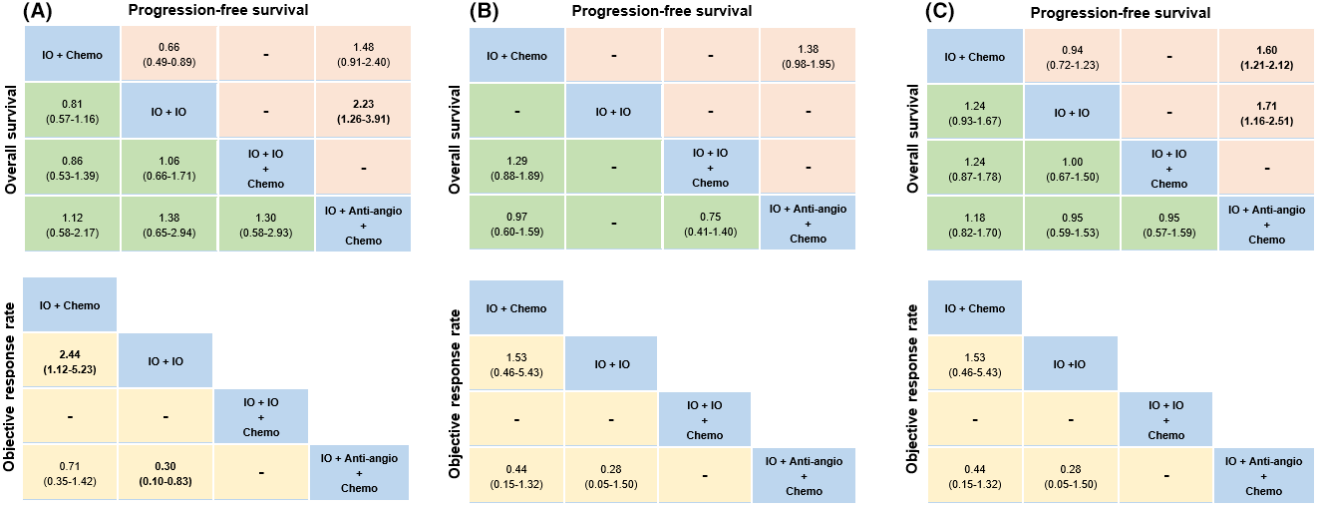
Pooled estimates of the network meta‐analysis according to histology. (A) PD‐L1‐high, (B) PD‐L1‐intermediate, and (C) PD‐L1‐negative cohort. Hazard ratios less than 1 and odds ratios more than 1 favor the former treatment. Significant results are in bold. We did not show the analysis for Anti‐angio + Chemo and Chemo. IO + Anti‐angio + Chemo; Immunotherapy + Anti‐angiogenic therapy + Chemotherapy; IO + Chemo, Immunotherapy + Chemotherapy; IO + IO; Immunotherapy + Immunotherapy; IO + IO + Chemo; Immunotherapy + Immunotherapy + Chemotherapy

In terms of OS (Figure 5A), no significant differences were detected among these four treatment modes. From the angle of PFS (Figure 5A), IO + Anti‐angio + Chemo performed significantly better than IO + IO and other differences were not detected among these modes. For ORR (Figure 5A), both IO + Chemo and IO + Anti‐angio + Chemo showed significantly higher ORR than IO + IO. We found IO + Anti‐angio + Chemo was most likely to be ranked first for OS, PFS, and ORR (probability = 58%; 94%; 81%) in PD‐L1‐high cohort according to the ranking profiles (Figure S3). The detailed probability distribution of ranking is shown in Table S4a.

##### PD‐L1‐intermediate cohort

There were no significant differences in these comparable treatment modes for OS, PFS, and ORR (Figure 5B). According to Bayesian ranking profiles (Figure S4), we found that IO + IO + Chemo tended to be the most preferable mode for OS (probability = 78%), IO + Anti‐angio + Chemo was most likely to be ranked first for PFS (probability = 96%) and ORR (probability = 95%). The detailed probability distribution of ranking is shown in Table S4b.

##### PD‐L1‐negative cohort

In terms of OS and ORR (Figure 5C), no significant differences were detected among these comparable treatment modes. From the ranking profiles (Figure S5), we found that IO + IO + Chemo and IO + Anti‐angio + Chemo had the highest possibility to increase OS (probability = 37%) and ORR (probability = 83%), respectively. For PFS (Figure 5C), IO + Chemo and IO + IO were inferior to IO + Chemo + Antiangio (HR = 1.60, 95% CI: 1.21–2.12; HR = 1.71, 95% CI: 1.16–2.51). Bayesian ranking profiles (Figure S5) suggested that IO + Chemo + Antiangio was most likely to be ranked first for PFS (probability = 100%). The detailed probability distribution of ranking is shown in Table S4c.

#### 3.3.2 Efficacy analysis of treatment modes according to histology

##### Non‐squamous cohort

**FIGURE 6:**
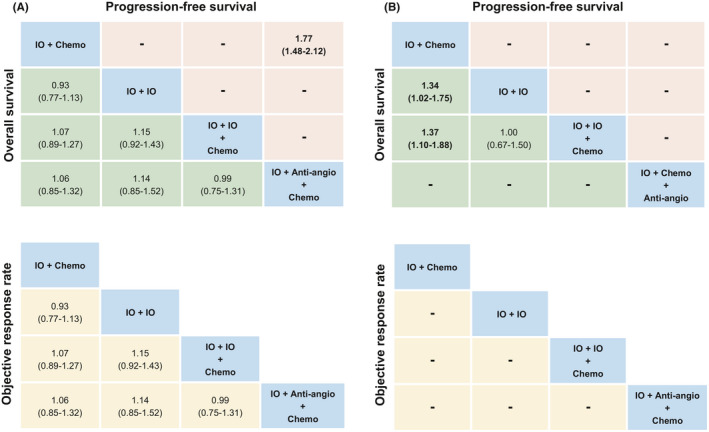
Pooled estimates of the network meta‐analysis according to PD‐L1 expression. (A) Non‐squamous cohort and (B) squamous cohort. Hazard ratios less than 1 and odds ratios more than 1 favor the former treatment. Significant results are in bold. We did not show the analysis for Anti‐angio + Chemo and Chemo. IO + Anti‐angio + Chemo; Immunotherapy + Anti‐angiogenic therapy + Chemotherapy; IO + Chemo; Immunotherapy + Chemotherapy; IO + IO; Immunotherapy + Immunotherapy; IO + IO + Chemo; Immunotherapy + Immunotherapy + Chemotherapy.

In terms of PFS (Figure 6A), IO + Anti‐angio + Chemo was superior to IO + Chemo significantly. Bayesian ranking profiles (Figure S6) indicated that IO + Anti‐angio + Chemo was most likely to be ranked first for PFS (probability = 95%). For OS and ORR (Figure 6A), there were no significant differences among these comparable treatment modes. Bayesian ranking profiles (Figure S6) suggested that IO + IO + Chemo was most likely to be ranked first to offer best OS (probability = 46%) and IO + Anti‐angio + Chemo tended to be ranked first to increase ORR (probability = 98%). The detailed probability distribution of ranking is shown in Table S5a.

##### Squamous cohort

In these comparable treatment modes, IO + IO and IO + IO + Chemo performed better OS (Figure 6B) than IO + Chemo significantly. Because of limitations of the included studies, we failed to get the PFS and ORR comparison results of each treatment mode. Bayesian ranking profiles (Figure S7) indicated that IO + IO and IO + IO + Chemo displayed same chance to be the optimal mode (probability = 50%). The detailed probability distribution of ranking is shown in Table S5b.

### 3.4 Heterogeneity and inconsistency assessment

Two feasible pair‐wise comparisons of forest plots with heterogeneity estimates are generated in Figures S8 and S9. Our evaluation showed that minimal (*I* ^2^ = 0%) or low heterogeneity was detected in all comparisons regarding OS and PFS in the whole population. Moreover, Figures S8 and S9 indicate that direct and indirect evidence were consistent, namely the estimates of PWMA were in concordance with that of NMA.

### 3.5 Sensitivity analysis

To assure the robustness and reliability of results, we conducted sensitivity analysis by removing studies with different study design with others. Given Ipi + Chemo is the only cytotoxic T‐lymphocyte‐associated protein 4 (CTLA‐4) inhibitor combined with chemotherapy in IO + Chemo mode, so we excluded this regimen to perform further analysis. Results of excluding Ipi + Chemo did not show obvious deviations compared with the original network meta‐analysis, probability of ranking was highly consistent (Figure S10).

## 4 DISCUSSION

Immune‐based combination therapies have been explored as first‐line treatment for advanced NSCLC patients. However, there is lack of systematic comparison among existing treatment modes. Our study provided a gauge to fill this gap and promote scientific practice of immune‐based combination therapies in advanced NSCLC. In this study, we found IO +IO + Chemo appears to be the best mode to improve OS and IO +Chemo + Antiangio may be the best possible mode for PFS and ORR with increasing TRAEs in general.

The combination of chemotherapy and immunotherapy is a pioneer in the era of immune‐based combination therapies. The addition of chemotherapy to immunotherapy leads to the immunogenic death (ICD) of lung cancer cells to promote the antitumor immune response, increase the presentation of tumor antigen, and improve the immunogenicity.^26^ The combination of CTLA‐4 and PD‐1 inhibitor can fully release the killing function of T cells and avoid the phenomenon of tumor escape in the initial stage of immunity and immune response stage.^27^ Based on the above, IO + IO + Chemo has a comprehensive antitumor effect and it can bring long‐term survival benefit. In terms of the indistinctive improvement in PFS and ORR of IO + IO + Chemo mode in our study, the possible explanation is that the failure of quick effect may be related to the delayed immune response, or to pseudoprogression, defined as of volume of tumor increasing caused by immune infiltration rather than tumor growth.^28^ Similar results that delayed benefit of immunotherapy were found in the treatment of melanoma with ipilimumab and renal cancer with nivolumab in some studies.^29^, ^30^, ^31^ It should be noted that ipilimumab and nivolumab are exactly the drugs used in IO + IO + Chemo mode. Notably, CCTG BR‐34 trial^32^ reported Durvalumab + Tremelimumab + Chemotherapy extended median PFS (7.7 months vs. 3.2 months, HR = 0.67, *p* = 0.0035) and improved ORR (27.7% vs. 14.1%, *p* = 0.001) in comparison with Durvalumab + Tremelimumab, but there was no significant difference in OS (16.6 months vs. 14.1 months, HR = 0.88, *p* = 0.46). Therefore, we expect more evidence of survival benefits of treatment regimens belonging to IO + IO + Chemo mode.

Compared with other modes, IO + Anti‐angio + Chemo mode performed greater advantages in improving the short‐term survival, namely PFS and ORR. Anti‐angio + Chemo has become the first‐line treatment for advanced non‐squamous NSCLC without sensitive gene mutations.^33^ Immunotherapy has synergistic effect not only with chemotherapy, but also with anti‐angiogenic therapy. Anti‐angiogenic drugs can promote vascular normalization by acting on immature blood vessels to reduce the activity of immunosuppressive cells such as myeloid‐derived suppressor cells (MDSCs), regulatory T cells (Tregs) which can reshape tumor microenvironment (TME). By blocking the inhibition of dendritic cell maturation mediated by vascular endothelial growth factor (VEGF), T cells can be activated more effectively. In addition, normalization of tumor vascular structure can promote the invasion of cytotoxic T lymphocytes (CTLs) into tumor.^34^ So IO + Anti‐angio + Chemo can take into account the antitumor effect from comprehensive aspects. However, we should pay attention to the significant adverse reactions associated with IO + Anti‐angio + Chemo according to the ranking profiles, which may explain why this mode cannot achieve significant long‐term survival benefit. In addition, there is short evidence of long‐term benefit from combination of anti‐angiogenic therapy and chemotherapy, while a lot of evidence have confirmed that immunotherapy can bring long‐term survival to patients. Therefore, compared with IO + IO + Chemo mode, the immune intensity of IO + Anti‐angio + Chemo mode is low, which may also be the reason for its lower long‐term survival rate. This mode may be able to be recommended in neoadjuvant therapy to achieve the effect of rapid phase‐down and tumor shrinkage and strive for more surgical opportunities for resectable NSCLC.

In the process of immunotherapy, efficacy and adverse reactions often occur together. Among all the adverse reactions, immune‐related adverse events (irAEs) are the most concerned. Because Chemo and Anti‐angio + Chemo could not transmit the comparison of irAEs in the network and some of the trials did not report irAEs, so we failed to include irAEs in the outcome analysis. But it is worth noting that among all treatment options of immunotherapy, combination of CTLA‐4 and PD‐1 inhibitor is most likely to cause irAEs, with an incidence of 55%–60%.^35^ On the other hand, many studies reported that development of irAEs predicted better outcomes in the process of immunotherapy.^36^, ^37^ Therefore, this can partly explain why IO + IO + Chemo was most likely to the best mode to improve OS. At present, irAEs of IO + Anti‐angio + Chemo mode in the real world remain to be studied, but it can be inferred from our research results that the incidence irAEs of IO + Anti‐angio + Chemo may be low and adverse reactions not related to immune may be dominant which cannot improve the long‐term survival.

Current evidence is inclined to demonstrate that there exists benefits to apply ICIs according to PD‐L1 expression,^38^ so we performed a detailed subgroup analysis according to PD‐L1 expression. IO + IO + Chemo was most likely to the best mode to prolong OS except in PD‐L1‐high cohort, in which IO + Anti‐angio + Chemo mode was most likely to be ranked first to offer best OS. Consistent with the whole population, IO + Anti‐angio + Chemo mode tended to achieve better PFS and ORR than other treatment modes with quite obvious grade ≥3 TRAEs in all subgroups. This result suggests that without considering the adverse reactions, IO + Anti‐angio + Chemo may be a wise choice to improve OS, PFS, and ORR of first‐line treatment for PD‐L1‐high advanced NSCLC patients. Furthermore, in some studies of kidney cancer and gastric cancer, IO + Anti‐angio + Chemo did show a positive correlation between PD‐L1 expression and efficacy.^39^, ^40^, ^41^, ^42^ The specific mechanism is worth further exploring.

Because of the huge heterogeneity of different subtypes of NSCLC and different responses to immunotherapy, we also performed a subgroup analysis according to histology types. In non‐squamous cohort, the results of OS, PFS, and ORR were consistent with the whole population. In squamous cohort, IO + IO and IO + IO + Chemo performed better OS than IO + Chemo significantly and they displayed same chance to have the highest probability to improve OS. Thus dual immunotherapy showed great advantages in patients with squamous NSCLC. Squamous NSCLC may benefit more from the addition of another ICIs than adenocarcinoma NSCLC for the higher tumor mutation burden (TMB), higher PD‐L1 expression, and more activated CD8^ + ^T cells in the TME. First of all, it was reported that the overall TMB of NSCLC was 8.0 mutations/megabase (Mb) and the TMB of squamous was significantly higher than that of adenocarcinoma NSCLC (*p* = 0.024).^43^ Second, previous studies have shown that the frequency of PD‐L1 expression in T cells of squamous and adenocarcinoma NSCLC was 56.2% and 39.9%, respectively.^44^, ^45^ What is more, squamous histology was an independent factor of high expression of PD‐L1.^46^, ^47^, ^48^ Third, Kinoshita et al.^49^ confirmed that insufficient activation of infiltrating CD8^+^ T cells and enrichment of Foxp3^+^ Tregs caused the immunosuppressive TME in non‐smokers with adenocarcinoma, which was the opposite of squamous cell carcinoma.^50^ The above factors are beneficial to immunotherapy.

Although IO + IO + Chemo appears to be the best mode to prolong OS, Pembro + Chemo seemed to bring best OS when specific to regimen. The antitumor mechanisms of PD‐L1 inhibitor (atezolizumab) belonging to IO + Chemo mode are not as optimized as that of PD‐1 inhibitor (pembrolizumab). Pembrolizumab, as a PD‐1 inhibitor, can block the binding of PD‐1 to its ligands (PD‐L1 and PD‐L2), while PD‐L1 inhibitor only inhibits the binding of PD‐1 to PD‐L1 and PD‐1/PD‐L2 axis is not blocked, which may inhibit the activation of T cells and induce immune system suppression.^51^ Notably, Jie Wang et al. first proposed the application of “Mirror Principle” in meta‐analysis and systematically evaluated the difference in efficacy and safety between PD‐1 inhibitors and PD‐L1 inhibitors. The results showed that PD‐1 inhibitors had better OS benefit than PD‐L1 inhibitors (HR = 0.75, 95% CI: 0.65–0.86).^52^ Therefore, without the limitations of other regimens of IO + Chemo mode, the superiority of therapeutic effect of Pembro + Chemo was reflected. In addition, Nivo + Beva + Chemo and Atezo + Beva + Chemo performed greater advantages in improving PFS and ORR with obvious adverse events, which were in line with the results of IO + Anti‐angio + Chemo mode.

As far as we know, this is the most comprehensive network meta‐analysis analyzing the efficacy and safety of immune‐based combination therapies in the first‐line treatment for advanced NSCLC patients. Besides, our study also included the latest researches relevant to first‐line treatment of advanced NSCLC, such as Carem + Chemo and Nivo + Beva + Chemo. Nevertheless, there were some limitations in our study. First, heterogeneity among included RCTs may be inevitable, which may lead to deviations of results of this study. Second, there existed insignificant statistical differences in this study and we had to turn to the ranking profiles to speculate the best treatment modes. Therefore, there was a certain degree of deviations between the results from the ranking profiles and the actual situations. Third, due to the limited data of subgroup analysis in some trials, not all treatment modes were compared in our subgroup analysis. Therefore, more head‐to‐head studies and retrospective studies of the real world involving various subgroups to compare immune‐based combination therapies from mode to regimen should be conducted so that clinicians can formulate precise therapy.

In short, our research fully displays the strong vitality of immune‐based combination therapies for advanced NSCLC and each mode has its own merits. It is reasonable for us to anticipate the powerful boost of immune‐based combination therapies to bring unprecedented survival benefits to patients in the real world.

## CONFLICT OF INTEREST

All authors declare no competing interest.

## Supporting information

Fig S1‐10

Table S1‐5

## Data Availability

Only publicly available data were used in this study, and data sources and handling of these data are described in Tables 1 and 2 and in Section 2, respectively. Further details are available from the corresponding author upon request.

## References

[1] Siegel RL , Miller KD , Jemal A . Cancer statistics, 2019. CA Cancer J Clin. 2019;69(1):7‐34. doi:10.3322/caac.21551 30620402

[2] Barta JA , Powell CA , Wisnivesky JP . Global epidemiology of lung cancer. Ann Glob Health. 2019;85(1): doi:10.5334/aogh.2419 PMC6724220 30741509

[3] Carlisle JW , Steuer CE , Owonikoko TK , Saba NF . An update on the immune landscape in lung and head and neck cancers. CA Cancer J Clin. 2020;70(6):505‐517. doi:10.3322/caac.21630 32841388

[4] Malhotra J , Jabbour SK , Aisner J . Current state of immunotherapy for non‐small cell lung cancer. Transl Lung Cancer Res. 2017;6(2):196‐211. doi:10.21037/tlcr.2017.03.01 28529902 PMC5420529

[5] Huang MY , Jiang XM , Wang BL , Sun Y , Lu JJ . Combination therapy with PD‐1/PD‐L1 blockade in non‐small cell lung cancer: strategies and mechanisms. Pharmacol Ther. 2020;219. doi:10.1016/j.pharmthera.2020.107694 32980443

[6] Paz‐Ares L , Vicente D , Tafreshi A , et al. A randomized, placebo‐controlled trial of pembrolizumab plus chemotherapy in patients with metastatic squamous NSCLC: protocol‐specified final analysis of KEYNOTE‐407. J Thorac Oncol. 2020;15(10):1657‐1669. doi:10.1016/j.jtho.2020.06.015 32599071

[7] Gadgeel S , Rodríguez‐Abreu D , Speranza G , et al. Updated analysis from KEYNOTE‐189: pembrolizumab or placebo plus pemetrexed and platinum for previously untreated metastatic nonsquamous non‐small‐cell lung cancer. J Clin Oncol. 2020;38(14):1505‐1517. doi:10.1200/jco.19.03136 32150489

[8] Nishio M , Barlesi F , Ball S , et al. Final efficacy results from IMpower132: first‐line atezolizumab + chemotherapy in patients with stage IV non‐squamous NSCLC. Ann Oncol. 2020;31:S1386‐S1387. doi:10.1016/j.annonc.2020.10.369

[9] Reck M , Peters S , Ramalingam S , et al. Nivolumab (N) + low‐dose ipilimumab (I) vs platinum‐doublet chemotherapy (Chemo) as first‐line (1L) treatment (TX) for advanced non‐small cell lung cancer (NSCLC): checkmate 227 part 1 final analysis. Oncology Research and Treatment. 2020;43:233‐234. doi:10.1159/000506491

[10] Reck M , Ciuleanu TE , Cobo M , et al. First‐line nivolumab (NIVO) + ipilimumab (IPI) combined with 2 cycles of platinum‐based chemotherapy (chemo) vs 4 cycles of chemo in advanced non‐small cell lung cancer (NSCLC): patient‐reported outcomes (PROs) from CheckMate 9LA. Ann Oncol. 2020;31:S1187‐S1188. doi:10.1016/j.annonc.2020.08.2292

[11] Socinski MA , Mok TS , Nishio M , et al. IMpower150 final analysis: efficacy of atezolizumab (atezo) + bevacizumab (bev) and chemotherapy in first‐line (1L) metastatic nonsquamous (nsq) non‐small cell lung cancer (NSCLC) across keysubgroups. Can Res. 2020;80(16 SUPPL): doi:10.1158/1538-7445.AM2020-CT216

[12] Ando K , Kishino Y , Homma T , et al. Nivolumab plus ipilimumab versus existing immunotherapies in patients with PD‐L1‐positive advanced non‐small cell lung cancer. A systematic review and network meta‐analysis. Cancers. 2020;12(7). doi:10.3390/cancers12071905 PMC7409193 32679702

[13] Liu J , Li C , Seery S , Yu J , Meng X . Identifying optimal first‐line interventions for advanced non‐small cell lung carcinoma according to PD‐L1 expression: a systematic review and network meta‐analysis. Oncoimmunology. 2020;9(1):1746112. doi:10.1080/2162402x.2020.1746112 32313723 PMC7153822

[14] Liang H , Lin G , Wang W , et al. Feasibility and safety of PD‐1/L1 inhibitors for non‐small cell lung cancer in front‐line treatment: a Bayesian network meta‐analysis. Transl Lung Cancer Res. 2020;9(2):188‐203. doi:10.21037/tlcr.2020.02.14 32420059 PMC7225152

[15] Hutton B , Salanti G , Caldwell DM , et al. The PRISMA extension statement for reporting of systematic reviews incorporating network meta‐analyses of health care interventions: checklist and explanations. Ann Intern Med. 2015;162(11):777‐784. doi:10.7326/m14-2385 26030634

[16] Higgins JP , Altman DG , Gøtzsche PC , et al. The Cochrane Collaboration's tool for assessing risk of bias in randomised trials. BMJ. 2011;343:d5928. doi:10.1136/bmj.d5928 22008217 PMC3196245

[17] Jotte R , Cappuzzo F , Vynnychenko I , et al. Atezolizumab in combination with carboplatin and nab‐paclitaxel in advanced squamous NSCLC (IMpower131): results from a randomized phase III trial. J Thorac Oncol. 2020;15(8):1351‐1360. doi:10.1016/j.jtho.2020.03.028 32302702

[18] West H , McCleod M , Hussein M , et al. Atezolizumab in combination with carboplatin plus nab‐paclitaxel chemotherapy compared with chemotherapy alone as first‐line treatment for metastatic non‐squamous non‐small‐cell lung cancer (IMpower130): a multicentre, randomised, open‐label, phase 3 trial. Lancet Oncol. 2019;20(7):924‐937. doi:10.1016/s1470-2045(19)30167-6 31122901

[19] Paz‐Ares L , Ciuleanu TE , Yu X , et al. LBA3 Nivolumab (NIVO) + platinum‐doublet chemotherapy (chemo) vs chemo as first‐line (1L) treatment (tx) for advanced non‐small cell lung cancer (aNSCLC): CheckMate 227—part 2 final analysis. Ann Oncol. 2019;30:xi67‐xi68. doi:10.1093/annonc/mdz453.004

[20] Zhou C , Chen G , Huang Y , et al. A randomized phase 3 study of camrelizumab plus chemotherapy as 1st line therapy for advanced/metastatic non‐squamous non‐small cell lung cancer. World Conference on Lung Cancer. 2019;14:S215‐S216. doi:10.1016/S2213-2600(20)30365-9

[21] Borghaei H , Langer CJ , Gadgeel S , et al. 24‐month overall survival from KEYNOTE‐021 cohort G: pemetrexed and carboplatin with or without pembrolizumab as first‐line therapy for advanced nonsquamous non‐small cell lung cancer. J Thorac Oncol. 2019;14(1):124‐129. doi:10.1016/j.jtho.2018.08.004 30138764

[22] Lee JS , Sugawara S , Kang JH , et al. Randomized phase III trial of nivolumab in combination with carboplatin, paclitaxel, and bevacizumab as first‐line treatment for patients with advanced or recurrent non‐squamous NSCLC. Ann Oncol. 2020;31:S1184‐S1185. doi:10.1016/j.annonc.2020.08.2287

[23] Lu S , Yu Y , Yu X , et al. Tislelizumab + chemotherapy vs chemotherapy alone as first‐line treatment for locally advanced/metastatic nonsquamous NSCLC. Ann Oncol. 2020;31:S816‐S817. doi:10.1016/j.annonc.2020.08.1577

[24] Govindan R , Szczesna A , Ahn MJ , et al. Phase III trial of ipilimumab combined with paclitaxel and carboplatin in advanced squamous non‐small‐cell lung cancer. J Clin Oncol. 2017;35(30):3449‐3457. doi:10.1016/j.lungcan.2018.08.019 28854067

[25] Wang J , Lu S , Hu C , et al. Updated analysis of tislelizumab plus chemotherapy vs chemotherapy alone as first‐line treatment of advanced squamous non‐small cell lung cancer (SQ NSCLC). Ann Oncol. 2020;31:S817. doi:10.1016/j.annonc.2020.08.1578

[26] Song W , Shen L , Wang Y , et al. Synergistic and low adverse effect cancer immunotherapy by immunogenic chemotherapy and locally expressed PD‐L1 trap. Nat Commun. 2018;9(1):2237. doi:10.1038/s41467-018-04605-x 29884866 PMC5993831

[27] Havel JJ , Chowell D , Chan TA . The evolving landscape of biomarkers for checkpoint inhibitor immunotherapy. Nat Rev Cancer. 2019;19(3):133‐150. doi:10.1038/s41568-019-0116-x 30755690 PMC6705396

[28] Emens LA , Ascierto PA , Darcy PK , et al. Cancer immunotherapy: opportunities and challenges in the rapidly evolving clinical landscape. Eur J Cancer. 2017;81:116‐129. doi:10.1016/j.ejca.2017.01.035 28623775

[29] Hodi FS , O'Day SJ , McDermott DF , et al. Improved survival with ipilimumab in patients with metastatic melanoma. N Engl J Med. 2010;363(8):711‐723. doi:10.1056/NEJMoa1003466 20525992 PMC3549297

[30] Robert C , Thomas L , Bondarenko I , et al. Ipilimumab plus dacarbazine for previously untreated metastatic melanoma. N Engl J Med. 2011;364(26):2517‐2526. doi:10.1056/NEJMoa1104621 21639810

[31] Motzer RJ , Escudier B , McDermott DF , et al. Nivolumab versus everolimus in advanced renal‐cell carcinoma. N Engl J Med. 2015;373(19):1803‐1813. doi:10.1056/NEJMoa1510665 26406148 PMC5719487

[32] Kulkarni S , Laurie S , Goss G , et al. BR‐34‐randomized trial of durvalumab and tremelimumab +/‐ platinum chemotherapy in patients with metastatic squamous or non‐squamous NSCLC. J Thorac Oncol. 2018;13(10):S480‐S481. doi:10.1016/j.jtho.2018.08.608. 34800700

[33] Planchard D , Popat S , Kerr K , et al. Metastatic non‐small cell lung cancer: ESMO Clinical Practice Guidelines for diagnosis, treatment and follow‐up. Ann Oncol. 2018;29(Suppl 4) :iv192‐iv237. doi:10.1093/annonc/mdy275 30285222

[34] Fukumura D , Kloepper J , Amoozgar Z , Duda DG , Jain RK . Enhancing cancer immunotherapy using antiangiogenics: opportunities and challenges. Nat Rev Clin Oncol. 2018;15(5):325‐340. doi:10.1038/nrclinonc.2018.29 29508855 PMC5921900

[35] Shoushtari AN , Friedman CF , Navid‐Azarbaijani P , et al. Measuring toxic effects and time to treatment failure for nivolumab plus ipilimumab in melanoma. JAMA Oncol. 2018;4(1):98‐101. doi:10.1001/jamaoncol.2017.2391 28817755 PMC5833656

[36] Ahmed Y , Lee J , Calvert P . Thyroid related adverse events predict survival in NSCLC patients receiving anti‐PD‐1/ PD‐L1 therapy. J Thorac Oncol. 2019;14S(10):S930. doi:10.1016/j.jtho.2019.08.2020

[37] Berner F , Bomze D , Diem S , et al. Association of checkpoint inhibitor‐induced toxic effects with shared cancer and tissue antigens in non‐small cell lung cancer. JAMA Oncol. 2019;5(7):1043‐1047. doi:10.1001/jamaoncol.2019.0402 31021392 PMC6487908

[38] Gadgeel SM , Stevenson JP , Langer CJ , et al. Pembrolizumab and platinum‐based chemotherapy as first‐line therapy for advanced non‐small‐cell lung cancer: phase 1 cohorts from the KEYNOTE‐021 study. Lung Cancer. 2018;125:273‐281. doi:10.1016/j.lungcan.2018.08.019 30429032 PMC6886233

[39] Chau I , Bendell JC , Calvo E , et al. Interim safety and clinical activity in patients (pts) with advanced gastric or gastroesophageal junction (G/GEJ) adenocarcinoma from a multicohort phase 1 study of ramucirumab (R) plus pembrolizumab (P). J Clin Oncol. 2017;35(4_suppl):102. doi:10.1200/JCO.2017.35.4_suppl.102

[40] Chau I , Penel N , Soriano AO , et al. Ramucirumab in combination with pembrolizumab in treatment‐naïve advanced gastric or GEJ adenocarcinoma: safety and antitumor activity from the phase 1a/b JVDF trial. Cancers. 2020;12(10): doi:10.3390/cancers12102985 PMC7602637 33076423

[41] McDermott DF , Atkins MB , Motzer RJ , et al. A phase II study of atezolizumab (atezo) with or without bevacizumab (bev) versus sunitinib (sun) in untreated metastatic renal cell carcinoma (mRCC) patients (pts). J Clin Oncol. 2017;35:431. doi:10.1200/JCO.2017.35.6_suppl.431

[42] Grullich C , Motzer RJ , Powles T , et al. IMmotion151: a randomized phase III study of atezolizumab plus bevacizumab vs sunitinib in untreated metastatic Renal Cell Carcinoma (mRCC). Oncol Res Treatment. 2018;41:41‐42. doi:10.1159/000492737

[43] Chen Y , Liu Q , Chen Z , et al. PD‐L1 expression and tumor mutational burden status for prediction of response to chemotherapy and targeted therapy in non‐small cell lung cancer. J Exp Clin Cancer Res. 2019;38(1):193. doi:10.1186/s13046-019-1192-1 31088500 PMC6518807

[44] Yang CY , Lin MW , Chang YL , Wu CT , Yang PC . Programmed cell death‐ligand 1 expression is associated with a favourable immune microenvironment and better overall survival in stage I pulmonary squamous cell carcinoma. Eur J Cancer. 2016;57:91‐103. doi:10.1016/j.ejca.2015.12.033 26901614

[45] Yang CY , Lin MW , Chang YL , Wu CT , Yang PC . Programmed cell death‐ligand 1 expression in surgically resected stage I pulmonary adenocarcinoma and its correlation with driver mutations and clinical outcomes. Eur J Cancer. 2014;50(7):1361‐1369. 10.1016/j.ejca.2014.01.018 24548766

[46] Igawa S , Sato Y , Ryuge S , et al. Impact of PD‐L1 expression in patients with surgically resected non‐small‐cell lung cancer. Oncology. 2017;92(5):283‐290. doi:10.1159/000458412 28222447

[47] Cao L , Wang X , Li S , et al. PD‐L1 is a Prognostic biomarker in resected NSCLC patients with moderate/high smoking history and elevated serum SCCA level. J Cancer. 2017;8(16):3251‐3260. doi:10.7150/jca.21118 29158797 PMC5665041

[48] Inamura K , Yokouchi Y , Kobayashi M , et al. Tumor B7–H3 (CD276) expression and smoking history in relation to lung adenocarcinoma prognosis. Lung Cancer. 2017;103:44‐51. doi:10.1016/j.lungcan.2016.11.013 28024695

[49] Kinoshita T , Kudo‐Saito C , Muramatsu R , et al. Determination of poor prognostic immune features of tumour microenvironment in non‐smoking patients with lung adenocarcinoma. Eur J Cancer. 2017;86:15‐27. doi:10.1016/j.ejca.2017.08.026 28950145

[50] Schneider T , Kimpfler S , Warth A , et al. Foxp3(+) regulatory T cells and natural killer cells distinctly infiltrate primary tumors and draining lymph nodes in pulmonary adenocarcinoma. J Thorac Oncol. 2011;6(3):432‐438. doi:10.1097/JTO.0b013e31820b80ca 21258248

[51] Chen L , Han X . Anti‐PD‐1/PD‐L1 therapy of human cancer: past, present, and future. J Clin Invest. 2015;125(9):3384‐3391. doi:10.1172/jci80011 26325035 PMC4588282

[52] Duan J , Cui L , Zhao X , et al. Use of immunotherapy with programmed cell death 1 vs programmed cell death ligand 1 inhibitors in patients with cancer: a systematic review and meta‐analysis. JAMA Oncol. 2020;6(3):375‐384. doi:10.1001/jamaoncol.2019.5367 31876895 PMC6990765

