# Supplementary material for: First‐line immune‐based combination therapies for advanced non‐small cell lung cancer: A Bayesian network meta‐analysis
Source: Cancer Med. 2021 Nov 7;10(24):9139–55. doi: 10.1002/cam4.4405 (PMC8683544; doi:10.1002/cam4.4405)
Supplement: Supplementary file 1 — Fig S1‐10 [file CAM4-10-9139-s001.docx]

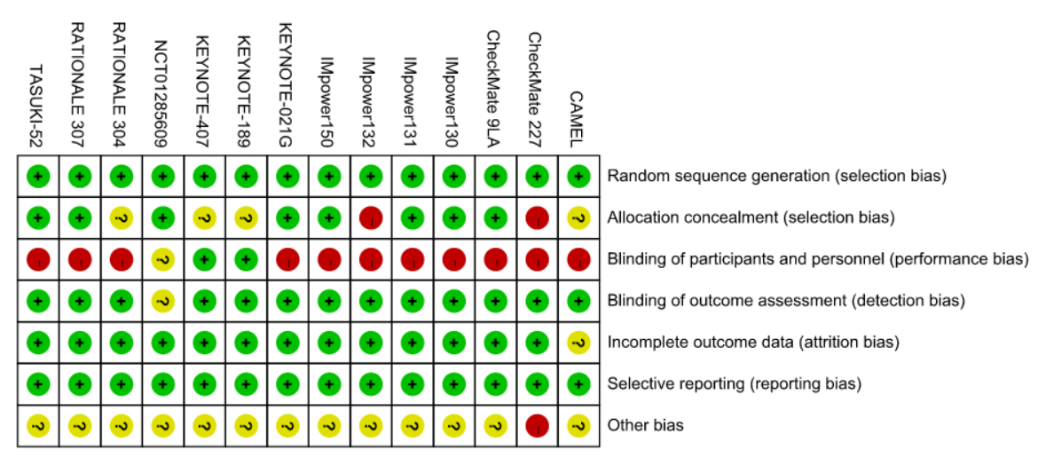

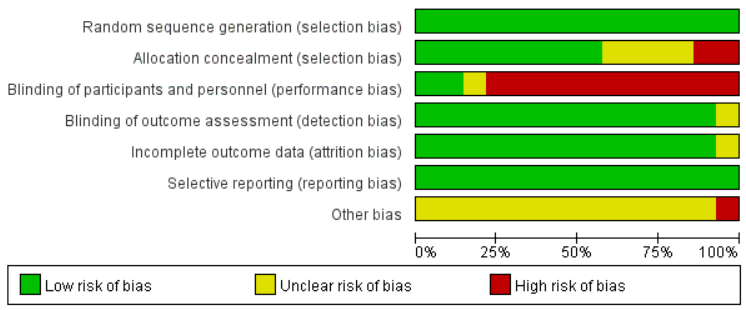


**Fig. S1** Quality assessment: risk of bias according to Cochrane Collaboration’s tool.

**Fig. S2** Bayesian ranking profiles of comparable treatment regimens on efficacy and safety for the whole population.

**Fig. S3** Bayesian ranking profiles of comparable treatment modes on efficacy for PD-L1-high cohort.

**Fig. S4** Bayesian ranking profiles of comparable treatment modes on efficacy for PD-L1-intermediate cohort.

**Fig. S5** Bayesian ranking profiles of comparable treatment modes on efficacy for PD-L1-negative cohort.

**Fig. S6** Bayesian ranking profiles of comparable treatment modes on efficacy for non-squamous cohort.

**Fig. S7** Bayesian ranking profiles of comparable treatment modes on efficacy for squamous cohort.


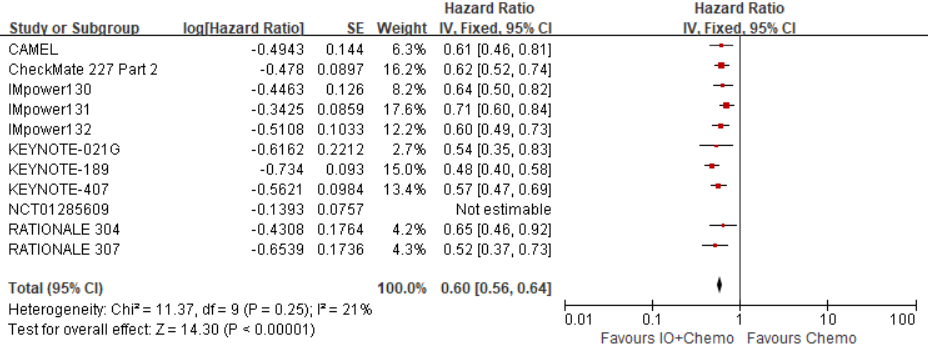

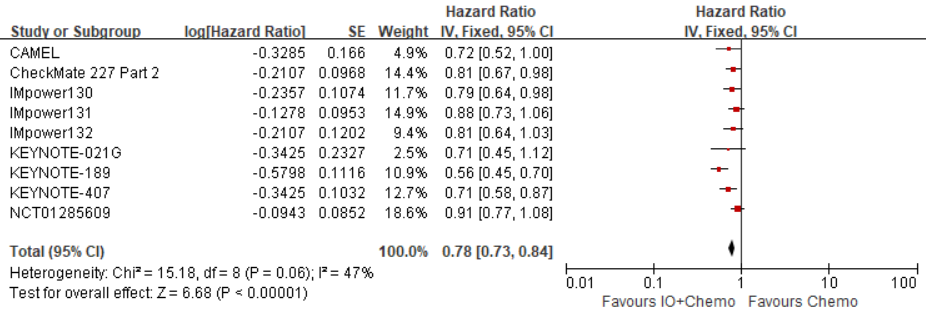


**
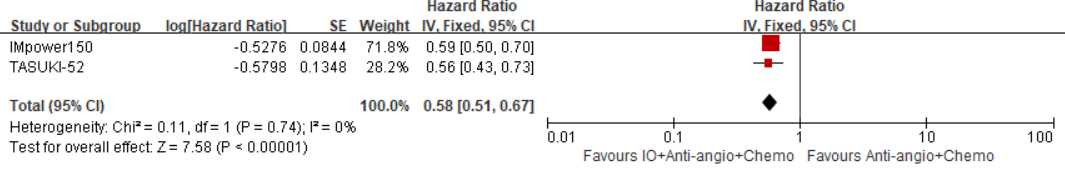
**
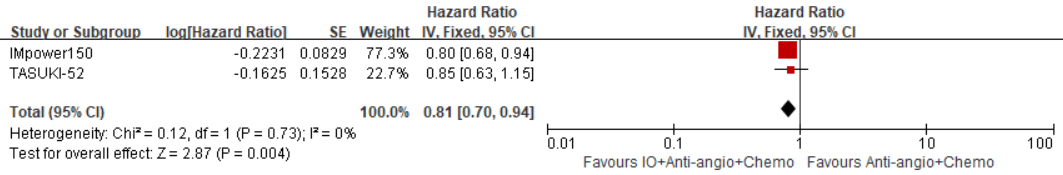


**Fig. S8** Forest plots for results of pairwise meta-analysis for overall survival in the whole population.

**Fig. S9** Forest plots for results of pairwise meta-analysis for progression-free survival in the whole population.

**Progression-free survival**

**Overall survival**

| **IO + Chemo** | **0.76**  **(0.65-0.89)** | 0.88  (0.72-1.07) | **1.56**  **(1.28-1.91)** |
| --- | --- | --- | --- |
| 1.04  (0.89-1.41) | **IO + IO** | 1.16  (0.92-1.47) | **2.06**  **(1.60-2.66)** |
| 1.15  (0.94-1.89) | 1.11  (0.88-1.40) | **IO + IO**  **+**  **Chemo** | **1.77**  **(1.34-2.36)** |
| 1.06  (0.85-1.32) | 1.02  (0.78-1.33) | 0.92  (0.68-1.24) | **IO + Anti-angio**  **+**  **Chemo** |

| **IO + Chemo** | 1.91  (0.94-3.92) | 1.16  (0.56-2.44) | 0.60  (0.26-1.39) |
| --- | --- | --- | --- |
| 1.64  (0.74-3.84) | **IO + IO** | 0.61  (0.23-1.59) | 0.31  (0.11-0.96) |
| 1.13  (0.48-2.65) | 0.69  (0.22-2.15) | **IO + IO**  **+**  **Chemo** | 0.52  (0.17-1.66) |
| 0.58  (0.22-1.57) | 0.36  (0.10-1.29) | 0.52  (0.14-1.95) | **IO + Anti-angio**  **+**  **Chemo** |

**Objective response rate**

**Grade ≥3 adverse treatment-related events**

**Fig. S10** Efficacy and safety analysis for treatment modes excluding “ Ipi + Chemo ” for the whole population.
