# Supplementary material for: First‐line immune‐based combination therapies for advanced non‐small cell lung cancer: A Bayesian network meta‐analysis
Source: Cancer Med. 2021 Nov 7;10(24):9139–55. doi: 10.1002/cam4.4405 (PMC8683544; doi:10.1002/cam4.4405)
Supplement: Supplementary file 2 — Table S1‐5 [file CAM4-10-9139-s002.docx]

**Table S1** Literature search criteria.

| **PubMed Search:**  ((((((((non-small-cell lung cancer[Title/Abstract]) OR (non-small cell lung cancer[Title/Abstract])) OR (non small-cell lung cancer[Title/Abstract])) OR (non small cell lung cancer[Title/Abstract])) OR (non-small-cell lung carcinoma[Title/Abstract])) OR (non-small cell lung carcinoma[Title/Abstract])) OR (non small-cell lung carcinoma[Title/Abstract])) OR (non small cell lung carcinoma[Title/Abstract])) OR (nsclc[Title/Abstract])) AND ((((((((((((((((((((((((((((((((((((((((((((((((((((((((pembrolizumab[Title/Abstract]) OR (lambrolizumab[Title/Abstract])) OR (Keytruda[Title/Abstract])) OR (MK-3475[Title/Abstract])) OR (nivolumab[Title/Abstract])) OR (MDX-1106[Title/Abstract])) OR (ONO-4538[Title/Abstract])) OR (BMS-936558[Title/Abstract])) OR (Opdivo[Title/Abstract])) OR (atezolizumab[Title/Abstract])) OR (MPDL3280A[Title/Abstract])) OR (Tecentriq[Title/Abstract])) OR (RG7446[Title/Abstract])) OR (RG-7446[Title/Abstract])) OR (camrelizumab[Title/Abstract])) OR (SHR-1210[Title/Abstract])) OR (SHR-1210[Title/Abstract])) OR (durvalumab[Title/Abstract])) OR (MEDI4736[Title/Abstract])) OR (MEDI-4736[Title/Abstract])) OR (Imfinzi[Title/Abstract])) OR (toripalimab[Title/Abstract])) OR (sintilimab[Title/Abstract])) OR (IBI 308[Title/Abstract])) OR (IBI308[Title/Abstract])) OR (IBI-308[Title/Abstract])) OR (tislelizumab[Title/Abstract])) OR (ipilimumab[Title/Abstract])) OR (Yervoy[Title/Abstract])) OR (MDX 010[Title/Abstract])) OR (MDX010[Title/Abstract])) OR (MDX-010[Title/Abstract])) OR (MDX-CTLA-4[Title/Abstract])) OR (MDX CTLA 4[Title/Abstract])) OR (immune checkpoint inhibitors[Title/Abstract])) OR (ICIs[Title/Abstract])) OR (immune checkpoint blockade[Title/Abstract])) OR (ICB[Title/Abstract])) OR (anti-PD1[Title/Abstract])) OR (PD-1[Title/Abstract])) OR (Programmed Death 1[Title/Abstract])) OR (Programmed Cell Death 1 Receptor[Title/Abstract])) OR (PD 1[Title/Abstract])) OR (PD1[Title/Abstract])) OR (Programmed Death-Ligand 1[Title/Abstract])) OR (PD-L1[Title/Abstract])) OR (programmed cell death 1 ligand 1 protein[Title/Abstract])) OR (PD L1[Title/Abstract])) OR (PDL1[Title/Abstract])) OR (CTLA-4[Title/Abstract])) OR (CD152[Title/Abstract])) OR (CTLA-4 Protein[Title/Abstract])) OR (CTLA 4 Protein[Title/Abstract])) OR (Cytotoxic T-Lymphocyte Antigen 4[Title/Abstract])) OR (Cytotoxic T Lymphocyte Antigen 4[Title/Abstract]) AND (((((((Randomized Controlled Trial[Publication Type]) OR (controlled clinical trial[Publication Type])) OR (randomized[Title/Abstract])) OR (randomised[Title/Abstract])) OR (randomly[Title/Abstract])) OR (trial[Title/Abstract])) OR (phase[Title/Abstract])) Filters: from 2015/1/1 - 2020/10/31  **966** |
| --- |
| **Embase Search:**  ('non small cell lung cancer'/exp OR (‘non-small-cell lung cancer’:ab,ti OR ‘non-small cell lung cancer’:ab,ti OR ‘non small-cell lung cancer’:ab,ti OR ‘non-small-cell lung carcinoma’:ab,ti OR ‘non-small cell lung carcinoma’:ab,ti OR ‘non small-cell lung carcinoma’:ab,ti OR ‘non small cell lung carcinoma’:ab,ti ‘nsclc’:ab,ti)) and (‘pembrolizumab’/exp OR ‘lambrolizumab’:ab,ti OR ‘Keytruda’:ab,ti OR ‘MK-3475’:ab,ti OR ‘nivolumab’/exp OR ‘MDX-1106’:ab,ti OR ‘ONO-4538’:ab,ti OR ‘BMS-936558’:ab,ti OR ‘Opdivo’:ab,ti OR ‘atezolizumab’/exp OR ‘anti-PDL1’:ab,ti OR ‘MPDL3280A’:ab,ti OR ‘Tecentriq’:ab,ti OR ‘RG7446’:ab,ti OR ‘RG-7446’:ab,ti OR ‘camrelizumab’:ab,ti OR ‘SHR-1210’:ab,ti OR ‘SHR 1210’:ab,ti OR ‘durvalumab’/exp OR ‘MEDI4736’:ab,ti OR ‘MEDI-4736’:ab,ti OR ‘Imfinzi’:ab,ti OR ‘MDX 010’:ab,ti OR ‘sintilimab’/exp OR ‘IBI 308’:ab,ti OR ‘IBI308’:ab,ti OR ‘IBI-308’:ab,ti OR ‘tislelizumab’:ab,ti OR ‘ipilimumab’/exp OR ‘Yervoy’:ab,ti OR ‘MDX010’:ab,ti OR ‘MDX-010’:ab,ti OR ‘MDX-CTLA-4’:ab,ti OR ‘MDX CTLA 4’/exp OR ‘immune checkpoint inhibitors’:ab,ti OR ‘ICIs’:ab,ti OR ‘immune checkpoint blockade’:ab,ti OR ‘ICB’:ab,ti OR ‘anti-PD1’:ab,ti OR ‘PD-1’:ab,ti OR ‘Programmed Death 1’:ab,ti OR ‘Programmed Cell Death 1 Receptor’:ab,ti OR ‘PD 1’:ab,ti OR ‘PD1’:ab,ti OR ‘Programmed Death-Ligand 1’:ab,ti OR ‘PD-L1’:ab,ti OR ‘programmed cell death 1 ligand 1 protein’:ab,ti OR ‘PD L1’:ab,ti OR ‘PDL1’:ab,ti OR ‘CTLA-4’:ab,ti OR ‘CD152’:ab,ti OR ‘CTLA-4 Protein’:ab,ti OR ‘CTLA 4 Protein’:ab,ti OR ‘Cytotoxic T-Lymphocyte Antigen 4’:ab,ti OR ‘Cytotoxic T Lymphocyte Antigen 4’:ab,ti AND (‘randomized controlled trial’/exp) AND (2015:py OR 2016:py OR 2017:py OR 2018:py OR 2019:py OR 2020:py)  **813** |
| #1 MeSH descriptor: [Carcinoma, Non-Small-Cell Lung] explode all trees  #2 "non-small cell lung cancer" OR "non small-cell lung cancer" OR "non small cell lung cancer" OR "non-small-cell lung carcinoma" OR "non-small cell lung carcinoma" OR "non small-cell lung carcinoma" OR "non small cell lung carcinoma"  #3 "pembrolizumab" OR "lambrolizumab" OR "Keytruda" or "MK-3475" OR "nivolumab" OR "MDX-1106" or "ONO-4538" OR "BMS-936558" OR "Opdivo" or "atezolizumab" OR "anti-PDL1" or "MPDL3280A" or "Tecentriq" or "RG7446" or "RG-7446" or “camrelizumab” OR “SHR-1210” OR “SHR 1210” OR “durvalumab” OR “MEDI4736” OR “MEDI-4736” OR “Imfinzi” OR “MDX 010” OR “sintilimab” OR “IBI 308” OR “IBI308” OR “IBI-308” OR “tislelizumab” OR “ipilimumab” OR “Yervoy” OR “MDX010” OR “MDX-010” OR ”MDX-CTLA-4” OR “MDX CTLA 4” OR “immune checkpoint inhibitors” OR “ICIs” OR “immune checkpoint blockade” OR “ICB” OR “anti-PD1” OR “PD-1” OR “Programmed Death 1” OR “Programmed Cell Death 1 Receptor” OR “PD 1” OR “PD1” OR “Programmed Death-Ligand 1” OR “PD-L1” OR “programmed cell death 1 ligand 1 protein” OR “PD L1” OR “PDL1” OR “CTLA-4” OR “CD152” OR “CTLA-4 Protein” OR “CTLA 4 Protein” OR “Cytotoxic T-Lymphocyte Antigen 4” OR “Cytotoxic T Lymphocyte Antigen 4”  # 4 (#1 OR #2) AND #3  **785** |

| Overall survival in whole population for treatment modes | | | | | | |
| --- | --- | --- | --- | --- | --- | --- |
| Treatment | Rank of possibility (%) | | | | | |
|  | 1 | 2 | 3 | 4 | 5 | 6 |
| IO + Chemo | 0 | 7 | **37** | 53 | 3 | 0 |
| IO + IO | 14 | **43** | 29 | 12 | 2 | 0 |
| IO + IO + Chemo | **68** | 22 | 7 | 3 | 0 | 0 |
| IO + Anti-angio + Chemo | 18 | 29 | 25 | **27** | 1 | 0 |
| Anti-angio + Chemo | 0 | 0 | 1 | 4 | **79** | 16 |
| Chemo | 0 | 0 | 0 | 0 | 16 | **84** |

| Progression-free survival in whole population for treatment modes | | | | | | |
| --- | --- | --- | --- | --- | --- | --- |
| Treatment | Rank of possibility (%) | | | | | |
|  | 1 | 2 | 3 | 4 | 5 | 6 |
| IO + Chemo | 0 | **80** | 19 | 1 | 0 | 0 |
| IO + IO | 0 | 0 | 3 | 17 | **80** | 0 |
| IO + IO + Chemo | 0 | 26 | **34** | 32 | 8 | 0 |
| IO + Anti-angio + Chemo | **100** | 0 | 0 | 0 | 0 | 0 |
| Anti-angio + Chemo | 0 | 9 | 30 | **48** | 13 | 0 |
| Chemo | 0 | 0 | 0 | 0 | 0 | **100** |

**Table S2**: Bayesian ranking results of network meta-analysis of different treatment modes in whole population. The number in each cell represents the posterior probability of the row-defining treatment being ranked at the columndefining position. The numbers represent the biggest probability of ranking first to last.

| Objective response rate in whole population for treatment modes | | | | | | |
| --- | --- | --- | --- | --- | --- | --- |
| Treatment | Rank of possibility (%) | | | | | |
|  | 1 | 2 | 3 | 4 | 5 | 6 |
| IO + Chemo | 6 | 30 | **42** | 20 | 2 | 0 |
| IO + IO | 2 | 4 | 8 | 18 | **44** | 24 |
| IO + IO + Chemo | 1 | 18 | 20 | **32** | 15 | 4 |
| IO + Anti-angio + Chemo | **80** | 12 | 5 | 2 | 1 | 0 |
| Anti-angio + Chemo | 1 | **38** | 24 | 24 | 10 | 3 |
| Chemo | 0 | 0 | 0 | 3 | 27 | **69** |

| Grade ≥3 adverse events in whole population for treatment modes | | | | | | |
| --- | --- | --- | --- | --- | --- | --- |
| Treatment | Rank of possibility (%) | | | | | |
|  | 1 | 2 | 3 | 4 | 5 | 6 |
| IO + Chemo | 4 | 10 | **62** | 23 | 1 | 0 |
| IO + IO | 0 | 1 | 2 | 8 | 22 | **67** |
| IO + IO + Chemo | 6 | 7 | 18 | **52** | 11 | 6 |
| IO + Anti-angio + Chemo | **79** | 13 | 4 | 2 | 1 | 0 |
| Anti-angio + Chemo | 10 | **68** | 13 | 7 | 1 | 0 |
| Chemo | 0 | 0 | 0 | 8 | **65** | 26 |

| Overall survival in whole population for treatment regimens | | | | | | | | | | | | |
| --- | --- | --- | --- | --- | --- | --- | --- | --- | --- | --- | --- | --- |
| Treatment | Rank of possibility (%) | | | | | | | | | | | |
|  | 1 | 2 | 3 | 4 | 5 | 6 | 7 | 8 | 9 | 10 | 11 | 12 |
| Pembro + Chemo | **39** | **33** | 17 | 7 | 3 | 1 | 0 | 0 | 0 | 0 | 0 | **-** |
| Atezo + Chemo | 0 | 0 | 0 | 3 | 12 | **24** | **32** | **22** | 6 | 0 | 0 | **-** |
| Carem + Chemo | 1 | 2 | 6 | 12 | 18 | 18 | 17 | 14 | 9 | 4 | 1 | **-** |
| Nivo + Chemo | 19 | 14 | 14 | 13 | 11 | 8 | 6 | 6 | 4 | 2 | 2 | **-** |
| Tisle + Chemo | **-** | **-** | **-** | **-** | **-** | **-** | **-** | **-** | **-** | **-** | **-** | **-** |
| Ipi + Chemo | 0 | 0 | 0 | 1 | 4 | 7 | 12 | 20 | **26** | 20 | 9 | **-** |
| Nivo + Ipi | 2 | 10 | **23** | **28** | **19** | 10 | 5 | 2 | 1 | 0 | 0 | **-** |
| Nivo + Ipi + Chemo | 29 | 29 | 20 | 12 | 6 | 3 | 1 | 0 | 0 | 0 | 0 | **-** |
| Atezo + Beva + Chemo | 4 | 7 | 11 | 15 | 18 | 16 | 12 | 10 | 5 | 2 | 0 | **-** |
| Nivo + Beva + Chemo | 5 | 5 | 7 | 9 | 11 | 12 | 11 | 12 | 11 | 8 | 9 | **-** |
| Beva + Chemo | 0 | 0 | 0 | 0 | 0 | 1 | 3 | 10 | 24 | **30** | 31 | **-** |
| Chemo | 0 | 0 | 0 | 0 | 0 | 0 | 0 | 3 | 14 | 34 | **49** | **-** |

**Table S3**: Bayesian ranking results of network meta-analysis of different treatment regimens in whole population. The number in each cell represents the posterior probability of the row-defining treatment being ranked at the columndefining position. The numbers represent the biggest probability of ranking first to last.

| Progression-free survival in whole population for treatment regimens | | | | | | | | | | | | |
| --- | --- | --- | --- | --- | --- | --- | --- | --- | --- | --- | --- | --- |
| Treatment | Rank of possibility (%) | | | | | | | | | | | |
|  | 1 | 2 | 3 | 4 | 5 | 6 | 7 | 8 | 9 | 10 | 11 | 12 |
| Pembro + Chemo | 2 | 7 | **57** | 26 | 7 | 1 | 0 | 0 | 0 | 0 | 0 | 0 |
| Atezo + Chemo | 0 | 0 | 0 | 3 | 12 | **26** | **34** | 21 | 3 | 0 | 0 | 0 |
| Carem + Chemo | 1 | 2 | 11 | 19 | 20 | 14 | 11 | 10 | 8 | 3 | 1 | 0 |
| Nivo + Chemo | 0 | 0 | 4 | 16 | **27** | 24 | 15 | 10 | 5 | 1 | 0 | 0 |
| Tisle + Chemo | 1 | 3 | 16 | **27** | 21 | 13 | 8 | 6 | 3 | 1 | 0 | 0 |
| Ipi + Chemo | 0 | 0 | 0 | 0 | 0 | 0 | 0 | 1 | 4 | 17 | **75** | 3 |
| Nivo + Ipi | 0 | 0 | 0 | 0 | 0 | 0 | 1 | 6 | 21 | **56** | 15 | 0 |
| Nivo + Ipi + Chemo | 0 | 0 | 0 | 4 | 9 | 15 | 19 | **24** | 21 | 6 | 1 | 0 |
| Atezo + Beva + Chemo | 39 | **53** | 6 | 2 | 0 | 0 | 0 | 0 | 0 | 0 | 0 | 0 |
| Nivo + Beva + Chemo | **58** | 34 | 5 | 2 | 1 | 0 | 0 | 0 | 0 | 0 | 0 | 0 |
| Beva + Chemo | 0 | 0 | 0 | 1 | 3 | 6 | 11 | 23 | **36** | 16 | 4 | 0 |
| Chemo | 0 | 0 | 0 | 0 | 0 | 0 | 0 | 0 | 0 | 0 | 3 | **97** |

| Objective response rate in whole population for treatment regimens | | | | | | | | | | | | |
| --- | --- | --- | --- | --- | --- | --- | --- | --- | --- | --- | --- | --- |
| Treatment | Rank of possibility (%) | | | | | | | | | | | |
|  | 1 | 2 | 3 | 4 | 5 | 6 | 7 | 8 | 9 | 10 | 11 | 12 |
| Pembro + Chemo | 15 | **23** | **23** | **17** | 10 | 7 | 2 | 1 | 1 | 0 | 0 | 0 |
| Atezo + Chemo | 0 | 0 | 1 | 2 | 6 | 12 | **24** | **31** | 19 | 4 | 1 | 0 |
| Carem + Chemo | 7 | 10 | 12 | 14 | 14 | **13** | 9 | 7 | 7 | 4 | 1 | 1 |
| Nivo + Chemo | 9 | 11 | 14 | **17** | **16** | **13** | 8 | 5 | 4 | 2 | 1 | 0 |
| Tisle + Chemo | 8 | 11 | 15 | 16 | **16** | 14 | 9 | 7 | 4 | 1 | 0 | 0 |
| Ipi + Chemo | 0 | 0 | 0 | 0 | 1 | 1 | 1 | 2 | 3 | 9 | 16 | **66** |
| Nivo + Ipi | 0 | 0 | 1 | 1 | 2 | 3 | 4 | 7 | 16 | **45** | 12 | 7 |
| Nivo + Ipi + Chemo | 2 | 3 | 5 | 7 | 10 | **13** | 16 | 15 | 18 | 8 | 2 | 2 |
| Atezo + Beva + Chemo | **43** | 19 | 12 | 8 | 7 | 4 | 3 | 2 | 1 | 1 | 0 | 0 |
| Nivo + Beva + Chemo | 15 | 22 | 13 | 12 | 11 | 10 | 7 | 4 | 3 | 2 | 1 | 1 |
| Beva + Chemo | 0 | 1 | 3 | 5 | 7 | 10 | 14 | 19 | **24** | 12 | 4 | 2 |
| Chemo | 0 | 0 | 0 | 0 | 0 | 0 | 0 | 1 | 3 | 13 | **60** | 23 |

| Grade ≥3 adverse events in whole population for treatment modes | | | | | | | | | | | | |
| --- | --- | --- | --- | --- | --- | --- | --- | --- | --- | --- | --- | --- |
| Treatment | Rank of possibility (%) | | | | | | | | | | | |
|  | 1 | 2 | 3 | 4 | 5 | 6 | 7 | 8 | 9 | 10 | 11 | 12 |
| Pembro + Chemo | 0 | 0 | 0 | 1 | 1 | 2 | 8 | 16 | **42** | 16 | 10 | 5 |
| Atezo + Chemo | 0 | 1 | 3 | 15 | **36** | **28** | 11 | 5 | 1 | 0 | 0 | 0 |
| Carem + Chemo | 17 | 21 | 17 | 23 | 9 | 6 | 3 | 2 | 1 | 0 | 0 | 0 |
| Nivo + Chemo | 1 | 2 | 3 | 6 | 13 | 17 | **25** | 23 | 7 | 2 | 1 | 1 |
| Tisle + Chemo | 2 | 5 | 7 | 10 | 17 | 23 | 17 | 12 | 4 | 1 | 1 | 0 |
| Ipi + Chemo | 0 | 0 | 0 | 0 | 1 | 1 | 2 | 4 | 12 | 13 | 23 | 44 |
| Nivo + Ipi | 0 | 0 | 0 | 0 | 0 | 1 | 1 | 2 | 7 | 10 | **34** | **45** |
| Nivo + Ipi + Chemo | 1 | 1 | 2 | 3 | 6 | 13 | 24 | **31** | 12 | 4 | 2 | 1 |
| Atezo + Beva + Chemo | **65** | 22 | 7 | 3 | 1 | 1 | 0 | 0 | 0 | 0 | 0 | 0 |
| Nivo + Beva + Chemo | 14 | **33** | 19 | 13 | 7 | 4 | 6 | 2 | 1 | 0 | 0 | 0 |
| Beva + Chemo | 1 | 16 | **35** | **31** | 10 | 4 | 2 | 1 | 0 | 0 | 0 | 0 |
| Chemo | 0 | 0 | 0 | 0 | 0 | 0 | 0 | 2 | 14 | **52** | 28 | 4 |

| Overall survival in PD-L1-high cohort for treatment modes | | | | | | |
| --- | --- | --- | --- | --- | --- | --- |
| Treatment | Rank of possibility (%) | | | | | |
|  | 1 | 2 | 3 | 4 | 5 | 6 |
| IO + Chemo | 25 | **45** | 23 | 6 | 1 | 0 |
| IO + IO | 4 | 12 | **25** | **34** | 26 | 0 |
| IO + IO + Chemo | 14 | 18 | 23 | 24 | 19 | 2 |
| IO + Anti-angio + Chemo | **58** | 15 | 11 | 11 | 3 | 1 |
| Anti-angio + Chemo | 0 | 10 | 18 | 4 | **79** | 16 |
| Chemo | 0 | 0 | 0 | 0 | 16 | **84** |

| Progression-free survival in PD-L1-high cohort for treatment modes | | | | | | |
| --- | --- | --- | --- | --- | --- | --- |
| Treatment | Rank of possibility (%) | | | | | |
|  | 1 | 2 | 3 | 4 | 5 | 6 |
| IO + Chemo | 6 | **93** | 1 | 0 | 0 | **-** |
| IO + IO | 0 | 1 | **58** | 42 | 0 | **-** |
| IO + IO + Chemo | **-** | **-** | **-** | **-** | **-** | **-** |
| IO + Anti-angio + Chemo | **94** | 6 | 0 | 0 | 0 | **-** |
| Anti-angio + Chemo | 0 | 1 | 41 | **56** | 2 | **-** |
| Chemo | 0 | 0 | 0 | 2 | **98** | **-** |

**Table S4**: Bayesian ranking results of network meta-analysis of different treatment modes according to PD-L1 expression. The number in each cell represents the posterior probability of the row-defining treatment being ranked at the columndefining position. The numbers represent the biggest probability of ranking first to last. (a) PD-L1-high, (b) PD-L1- intermediate and (c) PD-L1-negative cohort.

**a**

| Objective response rate in PD-L1-high cohort for treatment modes | | | | | | |
| --- | --- | --- | --- | --- | --- | --- |
| Treatment | Rank of possibility (%) | | | | | |
|  | 1 | 2 | 3 | 4 | 5 | 6 |
| IO + Chemo | 18 | **77** | 5 | 0 | 0 | **-** |
| IO + IO | 1 | 1 | 17 | **71** | 9 | **-** |
| IO + IO + Chemo | **-** | **-** | **-** | **-** | **-** | **-** |
| IO + Anti-angio + Chemo | **81** | 18 | 1 | 0 | 0 | **-** |
| Anti-angio + Chemo | 0 | 5 | **74** | 19 | 2 | **-** |
| Chemo | 0 | 0 | 0 | 12 | **88** | **-** |

| Overall survival in PD-L1-intermediate cohort for treatment modes | | | | | | |
| --- | --- | --- | --- | --- | --- | --- |
| Treatment | Rank of possibility (%) | | | | | |
|  | 1 | 2 | 3 | 4 | 5 | 6 |
| IO + Chemo | 5 | **52** | **39** | 4 | 0 | **-** |
| IO + IO | **-** | **-** | **-** | **-** | **-** | **-** |
| IO + IO + Chemo | **78** | 16 | 5 | 1 | 0 | **-** |
| IO + Anti-angio + Chemo | 17 | 30 | 30 | 18 | 4 | **-** |
| Anti-angio + Chemo | 0 | 1 | 6 | 35 | **48** | **-** |
| Chemo | 0 | 1 | 21 | **41** | 37 | **-** |

| Progression-free survival in PD-L1-intermediate cohort for treatment modes | | | | | | |
| --- | --- | --- | --- | --- | --- | --- |
| Treatment | Rank of possibility (%) | | | | | |
|  | 1 | 2 | 3 | 4 | 5 | 6 |
| IO + Chemo | 3 | **93** | 4 | 0 | **-** | **-** |
| IO + IO | **-** | **-** | **-** | **-** | **-** | **-** |
| IO + IO + Chemo | **-** | **-** | **-** | **-** | **-** | **-** |
| IO + Anti-angio + Chemo | **96** | 4 | 0 | 0 | **-** | **-** |
| Anti-angio + Chemo | 0 | 4 | **87** | 9 | **-** | **-** |
| Chemo | 0 | 0 | 9 | **91** | **-** | **-** |

**b**

| Objective response rate in PD-L1-intermediate cohort for treatment modes | | | | | | |
| --- | --- | --- | --- | --- | --- | --- |
| Treatment | Rank of possibility (%) | | | | | |
|  | 1 | 2 | 3 | 4 | 5 | 6 |
| IO + Chemo | 4 | **67** | 28 | 0 | **-** | **-** |
| IO + IO | **-** | **-** | **-** | **-** | **-** | **-** |
| IO + IO + Chemo | **-** | **-** | **-** | **-** | **-** | **-** |
| IO + Anti-angio + Chemo | **95** | 4 | 1 | 0 | **-** | **-** |
| Anti-angio + Chemo | 1 | 32 | **58** | 9 | **-** | **-** |
| Chemo | 0 | 1 | 9 | **90** | **-** | **-** |

| Overall survival in PD-L1-negative cohort for treatment modes | | | | | | |
| --- | --- | --- | --- | --- | --- | --- |
| Treatment | Rank of possibility (%) | | | | | |
|  | 1 | 2 | 3 | 4 | 5 | 6 |
| IO + Chemo | 0 | 3 | 22 | **40** | **35** | 0 |
| IO + IO | 34 | **36** | 19 | 7 | 4 | 0 |
| IO + IO + Chemo | **37** | 30 | 17 | 8 | 7 | 0 |
| IO + Anti-angio + Chemo | 28 | 25 | **28** | 13 | 5 | 1 |
| Anti-angio + Chemo | 0 | 10 | 18 | 22 | **35** | 14 |
| Chemo | 0 | 0 | 0 | 3 | 15 | 82 |

| Progression free survival in PD-L1-negative cohort for treatment modes | | | | | | |
| --- | --- | --- | --- | --- | --- | --- |
| Treatment | Rank of possibility (%) | | | | | |
|  | 1 | 2 | 3 | 4 | 5 | 6 |
| IO + Chemo | 0 | 13 | **61** | 26 | 0 | **-** |
| IO + IO | 0 | 15 | 20 | **64** | 1 | **-** |
| IO + IO + Chemo | **-** | **-** | **-** | **-** | **-** | **-** |
| IO + Anti-angio + Chemo | **100** | 0 | 0 | 0 | 0 | **-** |
| Anti-angio + Chemo | 0 | **72** | 19 | 9 | 0 | **-** |
| Chemo | 0 | 0 | 0 | 1 | **99** | **-** |

**c**

| Objective response rate in PD-L1- negative cohort for treatment modes | | | | | | |
| --- | --- | --- | --- | --- | --- | --- |
| Treatment | Rank of possibility (%) | | | | | |
|  | 1 | 2 | 3 | 4 | 5 | 6 |
| IO + Chemo | 3 | 21 | **63** | 13 | 0 | **-** |
| IO + IO | 4 | 7 | 13 | **47** | 29 | **-** |
| IO + IO + Chemo | **-** | **-** | **-** | **-** | **-** | **-** |
| IO + Anti-angio + Chemo | **83** | 12 | 3 | 1 | 1 | **-** |
| Anti-angio + Chemo | 11 | **59** | 19 | 8 | 4 | **-** |
| Chemo | 0 | 0 | 2 | 31 | **66** | **-** |

**Table S5**: Bayesian ranking results of network meta-analysis of different treatment modes according to histology. The number in each cell represents the posterior probability of the row-defining treatment being ranked at the columndefining position. The numbers represent the biggest probability of ranking first and last. (a) non-squamous cohort, (b) squamous cohort.

**a**

| Overall survival in non-squamous cohort for treatment modes | | | | | | |
| --- | --- | --- | --- | --- | --- | --- |
| Treatment | Rank of possibility (%) | | | | | |
|  | 1 | 2 | 3 | 4 | 5 | 6 |
| IO + Chemo | 6 | 31 | **46** | 16 | 1 | 0 |
| IO + IO | 4 | 12 | 18 | **40** | 25 | 0 |
| IO + IO + Chemo | **46** | **32** | 15 | 6 | 1 | 0 |
| IO + Anti-angio + Chemo | 44 | 25 | 17 | 14 | 0 | 0 |
| Anti-angio + Chemo | 0 | 1 | 4 | 23 | **66** | 6 |
| Chemo | 0 | 0 | 0 | 0 | 6 | **94** |

| Progression-free survival in non-squamous cohort for treatment modes | | | | | | |
| --- | --- | --- | --- | --- | --- | --- |
| Treatment | Rank of possibility (%) | | | | | |
|  | 1 | 2 | 3 | 4 | 5 | 6 |
| IO + Chemo | 0 | 32 | **68** | 0 | **-** | **-** |
| IO + IO | **-** | **-** | **-** | **-** | **-** | **-** |
| IO + IO + Chemo | **-** | **-** | **-** | **-** | **-** | **-** |
| IO + Anti-angio + Chemo | **100** | 0 | 0 | 0 | **-** | **-** |
| Anti-angio + Chemo | 0 | **68** | 32 | 0 | **-** | **-** |
| Chemo | 0 | 0 | 0 | **100** | **-** | **-** |

| Objective response rate in non-squamous cohort for treatment modes | | | | | | |
| --- | --- | --- | --- | --- | --- | --- |
| Treatment | Rank of possibility (%) | | | | | |
|  | 1 | 2 | 3 | 4 | 5 | 6 |
| IO + Chemo | 1 | **43** | 56 | 0 | **-** | **-** |
| IO + IO | **-** | **-** | **-** | **-** | **-** | **-** |
| IO + IO + Chemo | **-** | **-** | **-** | **-** | **-** | **-** |
| IO + Anti-angio + Chemo | **98** | 2 | 0 | 0 | **-** | **-** |
| Anti-angio + Chemo | 5 | **74** | **19** | 2 | **-** | **-** |
| Chemo | 0 | 0 | 12 | **88** | **-** | **-** |

| Overall survival in squamous cohort for treatment modes | | | | | | |
| --- | --- | --- | --- | --- | --- | --- |
| Treatment | Rank of possibility (%) | | | | | |
|  | 1 | 2 | 3 | 4 | 5 | 6 |
| IO + Chemo | 0 | 6 | **94** | 0 | - | - |
| IO + IO | **50** | **48** | 2 | 0 | - | - |
| IO + IO + Chemo | **50** | 46 | 4 | 0 | - | - |
| IO + Anti-angio + Chemo | - | - | - | - | - | - |
| Anti-angio + Chemo | - | - | - | - | - | - |
| Chemo | 0 | 0 | 0 | **100** | - | - |

| Progression-free survival in squamous cohort for treatment modes | | | | | | |
| --- | --- | --- | --- | --- | --- | --- |
| Treatment | Rank of possibility (%) | | | | | |
|  | - | - | - | - | - | - |
| IO + Chemo | - | - | - | - | - | - |
| IO + IO | - | - | - | - | - | - |
| IO + IO + Chemo | - | - | - | - | - | - |
| IO + Anti-angio + Chemo | - | - | - | - | - | - |
| Anti-angio + Chemo | - | - | - | - | - | - |
| Chemo | - | - | - | - | - | - |

**b**

| Objective response rate in squamous cohort for treatment modes | | | | | | |
| --- | --- | --- | --- | --- | --- | --- |
| Treatment | Rank of possibility (%) | | | | | |
|  | 1 | 2 | 3 | 4 | 5 | 6 |
| IO + Chemo | - | - | - | - | - | - |
| IO + IO | - | - | - | - | - | - |
| IO + IO + Chemo | - | - | - | - | - | - |
| IO + Anti-angio + Chemo | - | - | - | - | - | - |
| Anti-angio + Chemo | - | - | - | - | - | - |
| Chemo | - | - | - | - | - | - |
